# Supplementary material for: mTORC1 inhibition in cancer cells protects from glutaminolysis-mediated apoptosis during nutrient limitation
Source: Nat Commun. 2017 Jan 23;8:14124. doi: 10.1038/ncomms14124 (PMC5264013; doi:10.1038/ncomms14124)
Supplement: Supplementary Information — Supplementary Figures [file ncomms14124-s1.pdf]

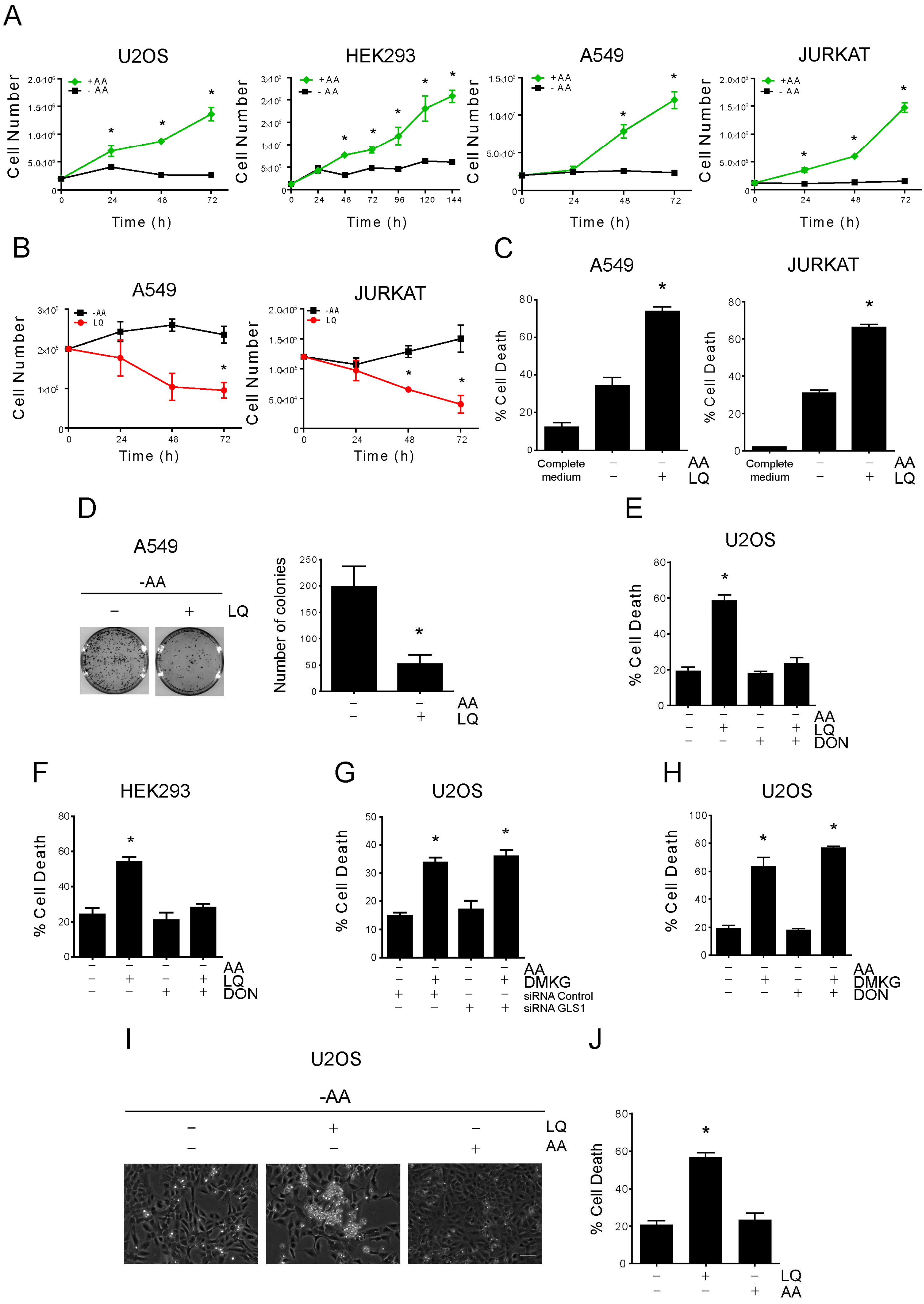

A
U2OS
HEK293
A549
JURKAT
Cell Number
Time (h)
+AA
- AA
B
A549
JURKAT
-AA
LQ
C
% Cell Death
Complete medium
AA
LQ
D
A549
-AA
LQ
Number of colonies
E
U2OS
DON
F
HEK293
G
DMKG
siRNA Control
siRNA GLS1
H
I
J

Supplementary Figure 1. Long-term glutaminolysis activation during amino acid restriction decreased cell viability. (A) Proliferation curves for the different cells lines (U2OS, HEK293, JURKAT, A549) upon amino acid deprivation (-AA) or amino acid sufficiency (+AA) for 24-144h. (**B**) Proliferation curve for A549 and JURKAT cells were measured upon amino acid starvation in the presence or absence of LQ after 24-72h. (**C**) The % of cell death was determined for the A549 and JURKAT cells after 72h for the conditions indicated. (**D**) A representative image of a clonogenic assay and the average number of the colonies are displayed for A549 cells. (**E, F**) % of cell death was measured upon amino acids starvation with or without LQ and the inhibitor of glutaminase, DON (40 µM) after 72h for U2OS (**E**) and after 144h HEK293 cells (**F**). (**G**) The % of cell death was determined for DMKG in cells depleted of GLS1 (siRNA GLS1) after 72h in U2OS cells (**H**) The % of cell death was determined for DMKG in the presence or absence of DON after 72h in U2OS cells. (**I**) Cells were starved (-AA) or not of all the amino acid (+AA) for 72h. A representative microscopy image for each condition is displayed. The scale bar represents 100 µm. (**J**) The % of cell death was measured for the conditions indicated in I. Graph shows the means ± SEM (n=3). * $p < 0.05$ (One-way Anova, post-hoc Bonferroni).

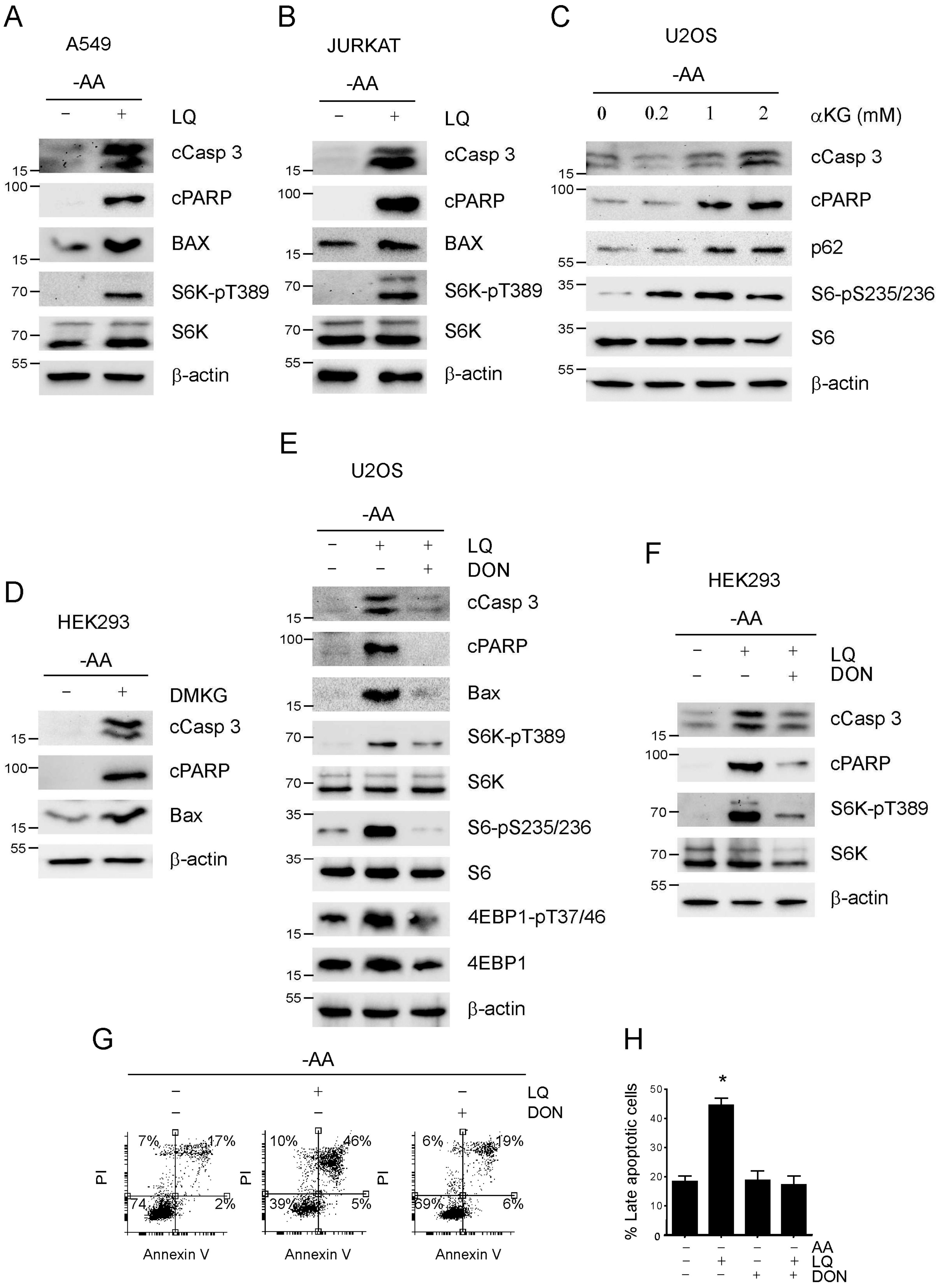

A
A549
-AA
LQ
cCasp 3
cPARP
BAX
S6K-pT389
S6K
β-actin
B
JURKAT
C
U2OS
0 0.2 1 2
αKG (mM)
p62
S6-pS235/236
S6
D
HEK293
DMKG
Bax
E
DON
4EBP1-pT37/46
4EBP1
F
G
PI
Annexin V
7% 17% 74 2%
10% 46% 39% 5%
6% 19% 69% 6%
H
% Late apoptotic cells
AA

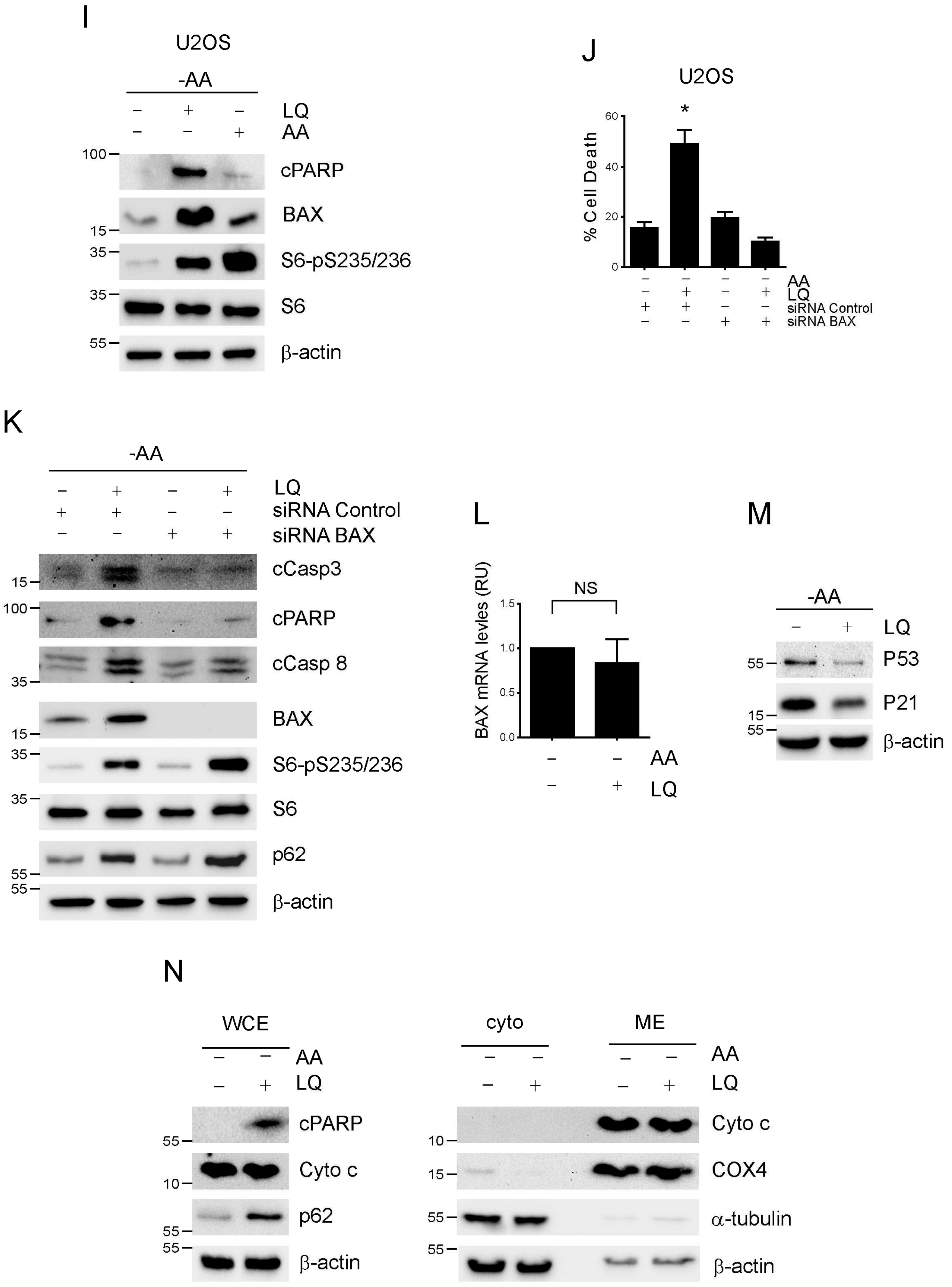
I
U2OS
-AA
LQ
AA
100
cPARP
BAX
15
35
S6-pS235/236
35
S6
55
β-actin
J
U2OS
% Cell Death
60
40
20
0
*
AA
LQ
siRNA Control
siRNA BAX
K
-AA
LQ
siRNA Control
siRNA BAX
cCasp3
15
100
cPARP
cCasp 8
35
BAX
15
35
S6-pS235/236
35
S6
p62
55
55
β-actin
L
BAX mRNA levles (RU)
1.5
1.0
0.5
0.0
NS
AA
LQ
M
-AA
LQ
55
P53
P21
15
55
β-actin
N
WCE
AA
LQ
cPARP
55
Cyto c
10
p62
55
55
β-actin
cyto
ME
AA
LQ
Cyto c
10
COX4
15
55
α-tubulin
55
β-actin

Supplementary Figure 2. Glutaminolysis activation during amino acid restriction induced apoptosis. (**A, B**) The activation of both mTORC1 and apoptotic markers were assessed for A549 and JURKAT cells after 72h for the conditions indicated. (**C**) Dose-dependent effect of DMKG (1, 0.2, 1, 2 mM) during amino acid starvation, on cell death, mTORC1 activity and autophagy (p62) in U2OS cells after 72h. (**D**) The activation of apoptotic markers were assessed in HEK293 cells after 144 h of DMKG treatment upon amino acid starvation. (**E**) The effect of glutaminolysis inhibition (DON) on LQ-induced apoptosis and mTORC1 activation in U2OS cells after 72h. (**F**) The effect of the inhibition of glutaminolysis (DON) on LQ-induced apoptosis and mTORC1 activation in HEK293 cells after 144 h. (**G**) Representative dot plot for Annexin V/PI staining of U2OS cells upon amino acid starvation in presence or absence of LQ and DON. (**H**) The % of late apoptosis (annexin V/PI positive cells) for the conditions indicated is displayed. (**I**) The effect of LQ or all the amino acids (+AA) on apoptosis and mTORC1 activity were assessed in U2OS cells after 72h. (**J**) The % of cell death was measured in cells depleted of BAX (siRNA BAX) upon amino acids starvation with or without LQ after 72h for U2OS. (**K**) The effect of knocking down BAX on apoptotic markers (cCaspase3, cPARP, cCaspase 8, BAX), mTORC1 activity (S6 phosphorylation) and autophagy (p62) induce by glutaminolysis. (**L**) U2OS cells were starved of amino acid in the presence or absence of LQ for 72h and the mRNA levels of BAX and RPL29 were measured. NS non-significant. (**M**) U2OS cells were starved of amino acid in the presence or absence of LQ for 72h and the expression of p53 and p21 were measured. (**N)** U2OS cells were starved of amino acid in the presence or absence of LQ for 72h and subjected to subcellular fractionation. In the whole cell extract (WCE) cPARP, p62, Cytochrome c and actin were assessed. In both the cytosolic and membrane fraction cytochrome c, COX4 (as a marker of membrane fraction), actin and tubulin (as markers of cytosolic fraction) were measured. Graph shows the means ± SEM (n=3). * $p < 0.05$ (One-way Anova, post-hoc Bonferroni).

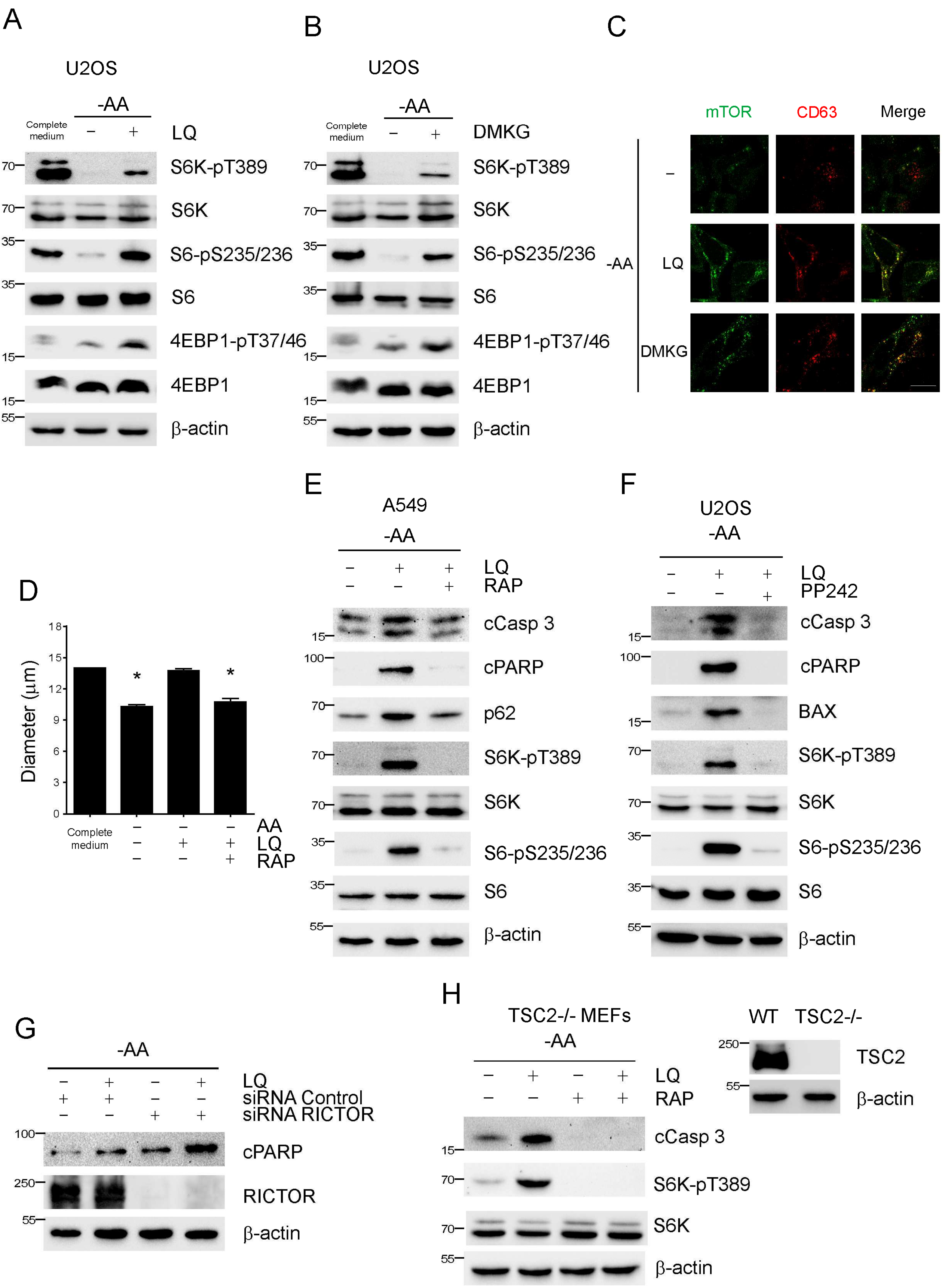
A
U2OS
-AA
Complete medium
−
+
LQ
S6K-pT389
S6K
S6-pS235/236
S6
4EBP1-pT37/46
4EBP1
β-actin
70
70
35
35
15
15
55
B
U2OS
-AA
Complete medium
−
+
DMKG
S6K-pT389
S6K
S6-pS235/236
S6
4EBP1-pT37/46
4EBP1
β-actin
70
70
35
35
15
15
55
C
mTOR
CD63
Merge
−
-AA
LQ
DMKG
D
Diameter (µm)
0
3
6
9
12
15
18
*
*
Complete medium
AA
LQ
RAP
E
A549
-AA
LQ
RAP
cCasp 3
cPARP
p62
S6K-pT389
S6K
S6-pS235/236
S6
β-actin
15
100
70
70
70
35
35
55
F
U2OS
-AA
LQ
PP242
cCasp 3
cPARP
BAX
S6K-pT389
S6K
S6-pS235/236
S6
β-actin
15
100
15
70
70
35
35
55
G
-AA
LQ
siRNA Control
siRNA RICTOR
cPARP
RICTOR
β-actin
100
250
55
H
TSC2-/- MEFs
-AA
LQ
RAP
cCasp 3
S6K-pT389
S6K
β-actin
15
70
70
55
WT
TSC2-/-
TSC2
β-actin
250
55

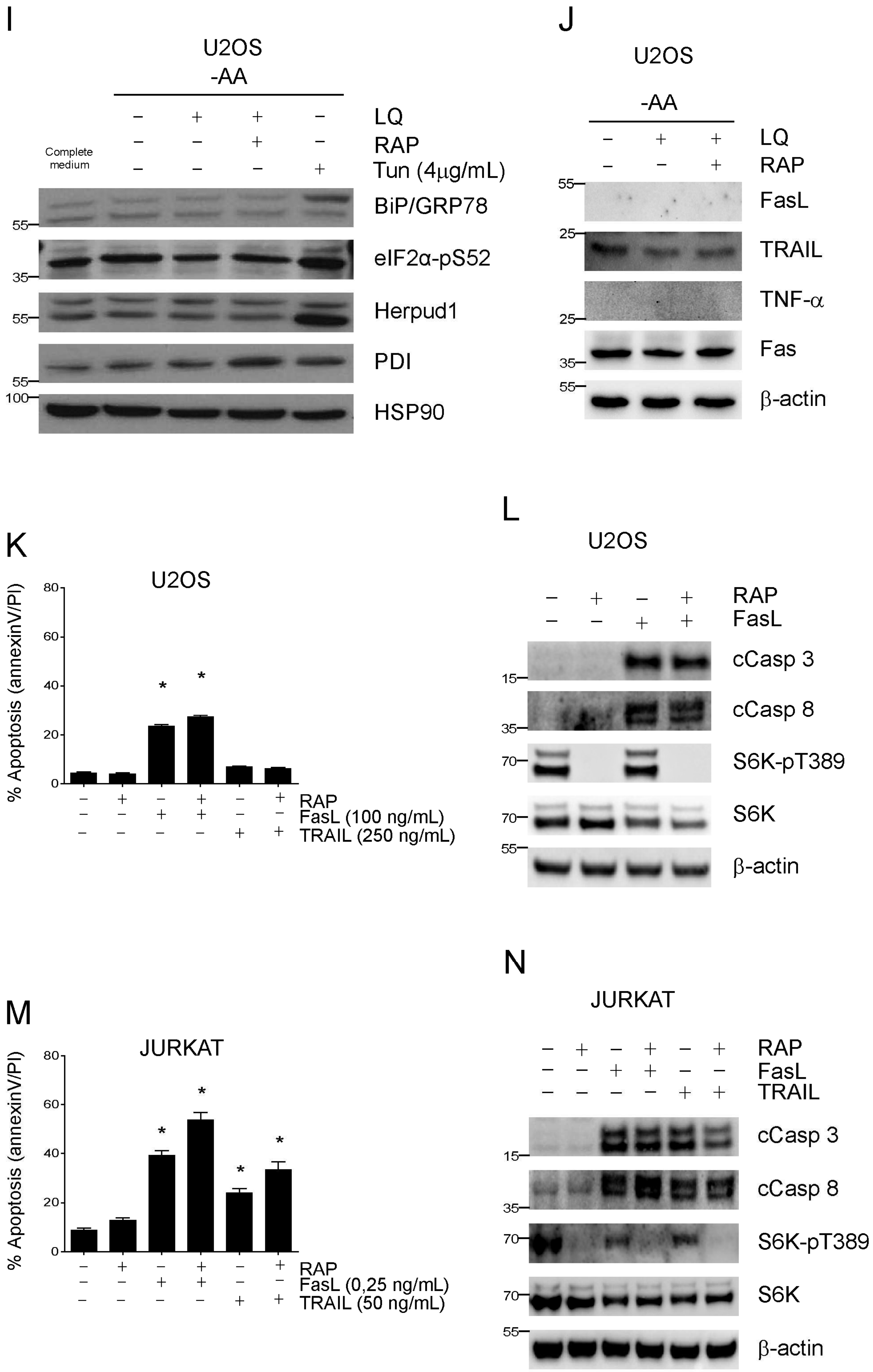
I
U2OS
-AA
LQ
RAP
Tun (4µg/mL)
Complete medium
BiP/GRP78
eIF2α-pS52
Herpud1
PDI
HSP90
55
35
55
55
100
J
U2OS
-AA
LQ
RAP
FasL
TRAIL
TNF-α
Fas
β-actin
55
25
25
35
55
K
U2OS
% Apoptosis (annexinV/PI)
80
60
40
20
0
RAP
FasL (100 ng/mL)
TRAIL (250 ng/mL)
L
U2OS
RAP
FasL
cCasp 3
cCasp 8
S6K-pT389
S6K
β-actin
15
35
70
70
55
M
JURKAT
% Apoptosis (annexinV/PI)
80
60
40
20
0
RAP
FasL (0,25 ng/mL)
TRAIL (50 ng/mL)
N
JURKAT
RAP
FasL
TRAIL
cCasp 3
cCasp 8
S6K-pT389
S6K
β-actin
15
35
70
70
55

Supplementary Figure 3. mTORC1 inhibition prevented the glutaminolysis-induced apoptosis. (**A**) Phosphorylation status of the downstream target of mTORC1 during amino acid starvation with or without LQ after 72h for U2OS cells. (**B**) Phosphorylation status of the downstream target of mTORC1 during amino acid starvation with or without DMKG after 72h for U2OS cells. (**C**) Colocalization of mTOR with CD63 (late lysosomal marker) for U2OS cells upon amino acid starvation with or without LQ or DMKG after 72h. (**D**) The cell size was measured for the U2OS cells after 72h for the conditions indicated. (**E**) The effect of the inhibition of mTORC1 (RAP) on glutaminolysis-induced apoptosis in A549 cells after 72h. (**F**) The effect of the inhibition of mTORC1/mTORC2 (PP242) on glutaminolysis-induced apoptosis in U2OS cells after 72h. (**G**) The effect of the genetic inhibition of mTORC2 (knock down RICTOR) on glutaminolysis-induced apoptosis. (**H**) Effect of glutaminolysis activation (LQ) upon amino acid starvation in TSC2-/- MEFs on apoptosis and mTORC1 activity after 72h. (**I**) The activation markers of ER stress and UPR were assessed in the conditions indicated after 72h. (**J**) The effect of glutaminolysis and rapamycin on the expression of the apoptosis-activating ligands, FasL, TRAIL, TNF-α, Fas were measured in U2OS cells after 72h. (**K**) The effects of FasL (100ng/mL) and TRAIL (250ng/mL) with or without rapamycin on the % apoptosis (annexin V/PI staining) after 24h in U2OS. (**L**) The effect FasL (100ng/mL) with or without rapamycin on apoptosis markers and mTORC1 downstream targets after 24h of treatment. (**M**) The effects of FasL (0,25ng/mL) and TRAIL (50ng/mL) with or without rapamycin on the % apoptosis (annexin V/PI staining) after 24h in JURKAT cells. (**M**) The effect of FasL (0,25ng/mL) and TRAIL (50ng/mL) with or without rapamycin on apoptosis markers and mTORC1 downstream targets after 24h of treatment in JURKAT cells. Graph bars show the means ± SEM (n=3). * $p < 0.05$ (One-way Anova, post-hoc Bonferroni).

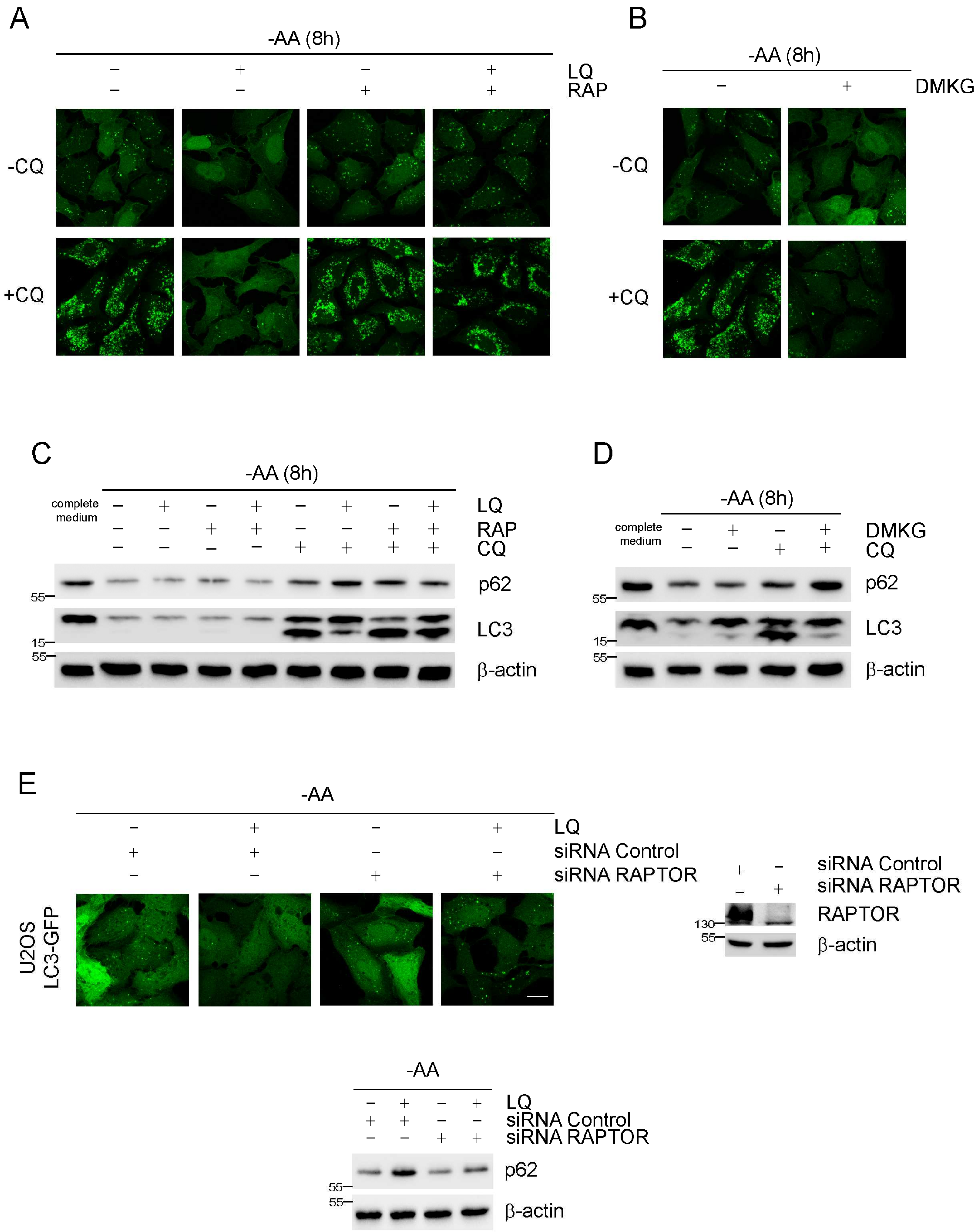
A
-AA (8h)
LQ
RAP
-CQ
+CQ
B
-AA (8h)
DMKG
-CQ
+CQ
C
-AA (8h)
complete medium
LQ
RAP
CQ
p62
LC3
β-actin
55
15
55
D
-AA (8h)
complete medium
DMKG
CQ
p62
LC3
β-actin
55
15
55
E
-AA
LQ
siRNA Control
siRNA RAPTOR
U2OS LC3-GFP
siRNA Control
siRNA RAPTOR
RAPTOR
β-actin
130
55
-AA
LQ
siRNA Control
siRNA RAPTOR
p62
β-actin
55
55

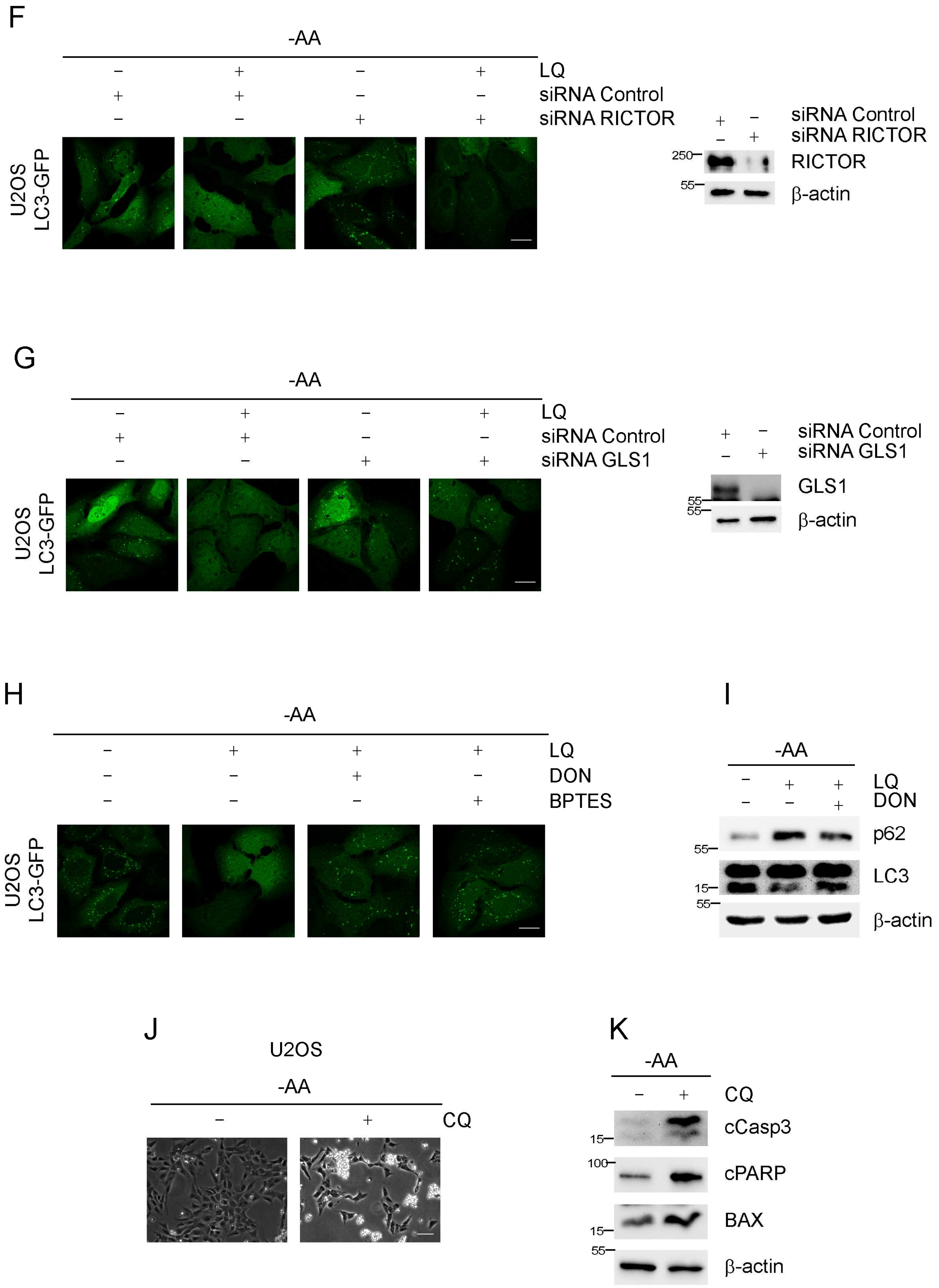
F
-AA
LQ
siRNA Control
siRNA RICTOR
U2OS LC3-GFP
siRNA Control
siRNA RICTOR
250
55
RICTOR
β-actin
G
-AA
LQ
siRNA Control
siRNA GLS1
U2OS LC3-GFP
siRNA Control
siRNA GLS1
55
55
GLS1
β-actin
H
-AA
LQ
DON
BPTES
U2OS LC3-GFP
I
-AA
LQ
DON
55
15
55
p62
LC3
β-actin
J
U2OS
-AA
CQ
K
-AA
CQ
15
100
15
55
cCasp3
cPARP
BAX
β-actin

Supplementary Figure 4. Glutaminolysis-activated cells showed an mTORC1-dependent inhibition of autophagy during amino acid restriction. (**A, B**) U2OS (GFP-LC3) were starved of amino acids in the present or absence of LQ, DMKG, RAP and with or without CQ for 8h. A representative confocal microscopy image for the condition indicated is displayed to evaluate autophagy flux. The scale bar represents 20 µm. (**C, D**) U2OS cells were starved of amino acids in the absence or presence of LQ, DMKG, RAP and CQ and the expression of p62 and LC3II was measured after 8h. U2OS GFP-LC3 cells were starved of amino acids with or without LQ for 72h in cells depleted of Raptor (**E**), Rictor (**F**) and GLS1 (**G**). Representative confocal images for these conditions are displayed. The siRNA Raptor and p62 levels, Rictor and GLS1 were validated by wester blot. (**H**) U2OS GFP-LC3 cells were starved of amino acid in the presence or absence of LQ, DON, BPTES for 72h. (**I**) The effect of the inhibition of glutaminolysis (DON) upon autophagy inhibition by LQ was assessed by the levels of p62 and LC3II. (**J, K**) The effect of CQ (10µM) on both the viability and the activation of apoptotic markers, upon amino acid starvation after 72h.

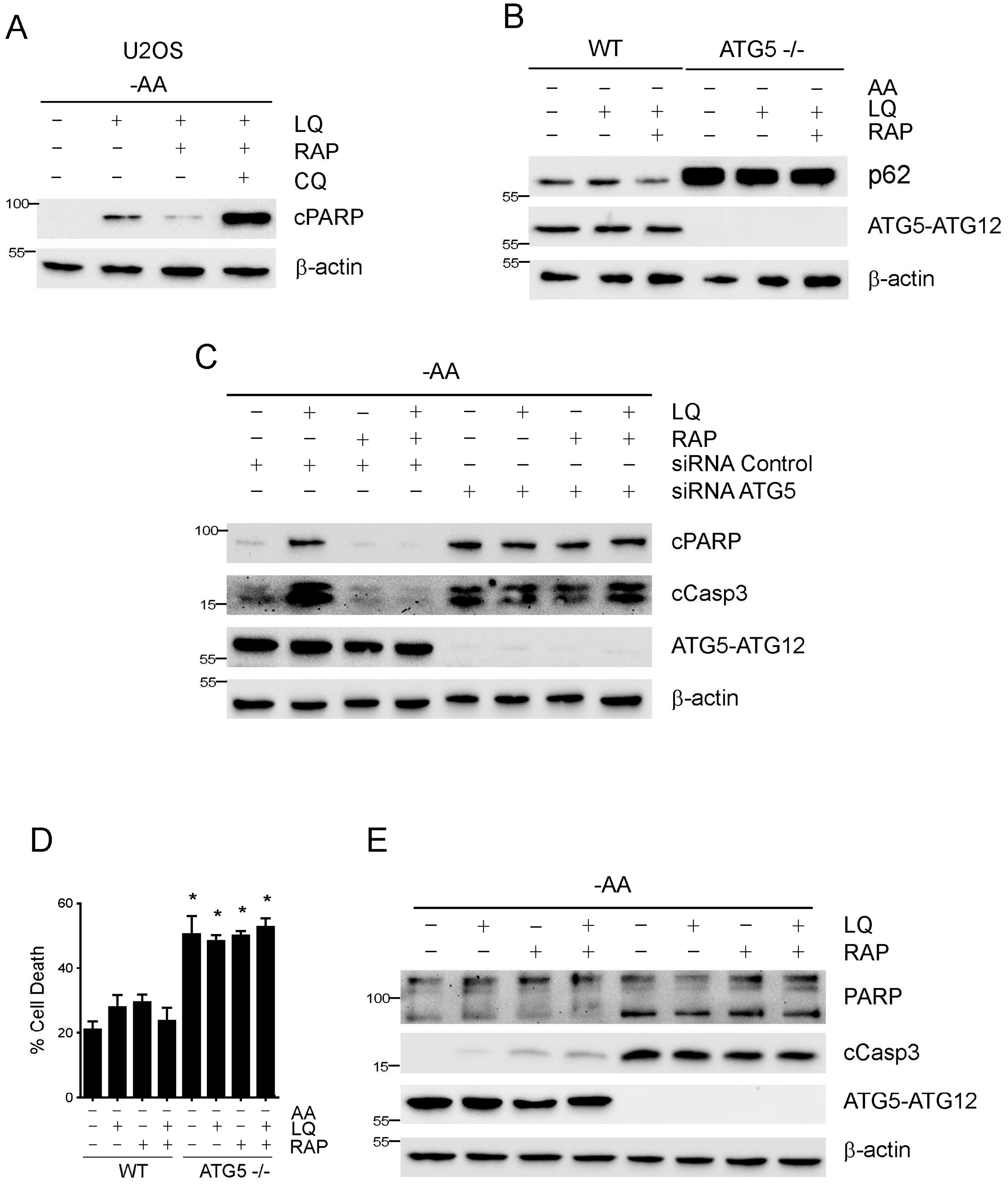
A
U2OS
-AA
LQ
RAP
CQ
100
cPARP
55
β-actin
B
WT
ATG5 -/-
AA
LQ
RAP
p62
55
ATG5-ATG12
55
55
β-actin
C
-AA
LQ
RAP
siRNA Control
siRNA ATG5
100
cPARP
cCasp3
15
ATG5-ATG12
55
55
β-actin
D
% Cell Death
60
40
20
0
AA
LQ
RAP
WT
ATG5 -/-
E
-AA
LQ
RAP
100
PARP
cCasp3
15
ATG5-ATG12
55
55
β-actin

Supplementary Figure 5. Autophagy was necessary for the ability of rapamycin treatment to prevent glutaminolysis/mTORC1-induced apoptosis apoptosis. (**A**) U2OS cells were starved of all the amino acids (-AA) in the presence or absence of LQ, RAP and CQ 10 μM for 72h and apoptotic marker cleaved PARP was measured. (**B**) WT and ATG5-/- MEFs were starved of amino acids in the presence or absence of LQ and RAP for 24h and autophagy was assessed by the levels of p62. (**C**) The apoptotic markers were measured in ATG5 depleted cells during amino acid starvation with or without LQ and RAP after 72h. (**D, E**) WT and ATG5-/- MEFs were starved of all amino acids (-AA) in the presence or absence of LQ and RAP for 24h, viability (**D**) and the cell death was assessed by the apoptotic markers (PARP and cCaspase 3, **E**). Graph bars show the means ± SEM (n=3). * $p < 0.05$ (One-way Anova, post-hoc Bonferroni).

Figure 1F

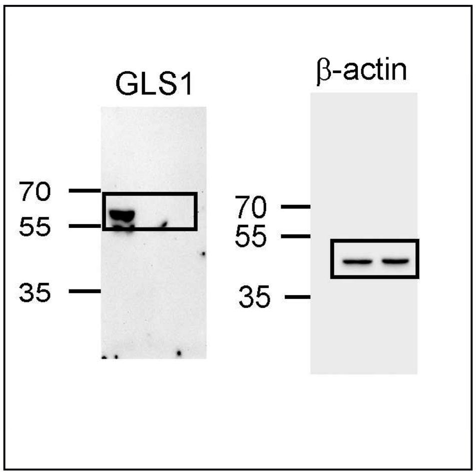

Figure 1G

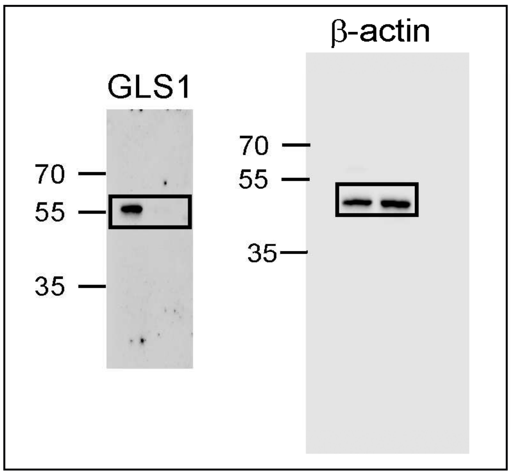

Figure 2A

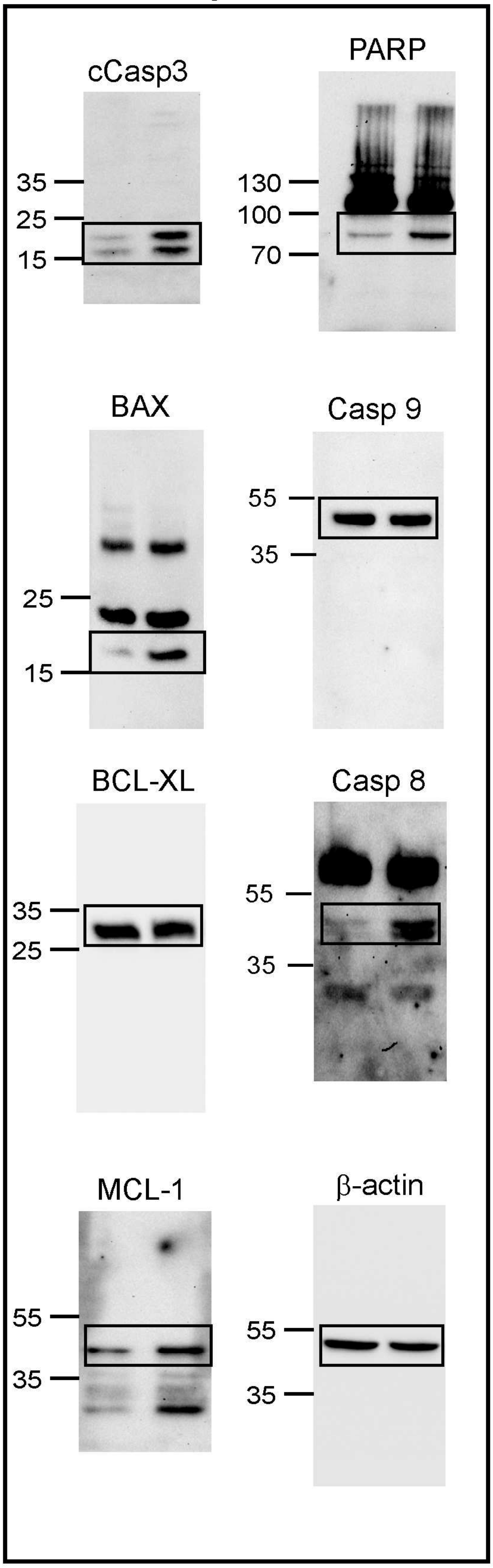

Figure 2B

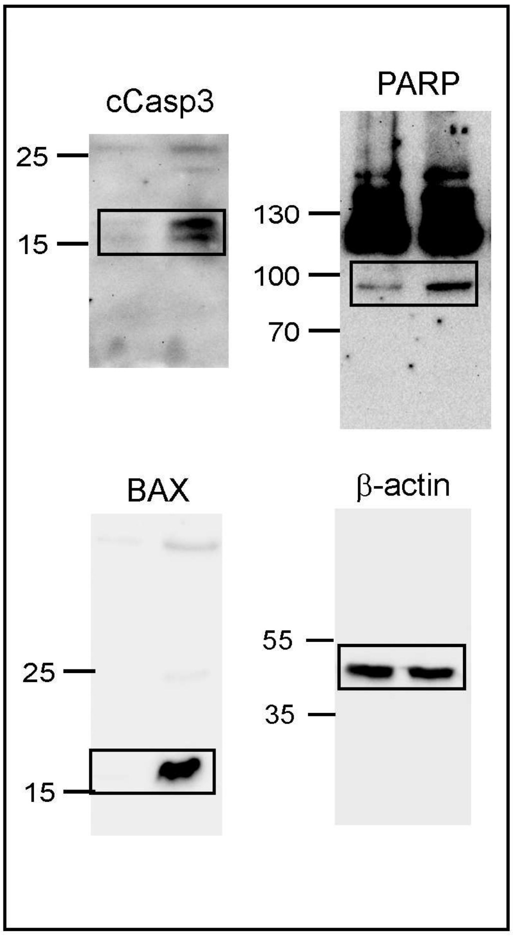

Figure 2G

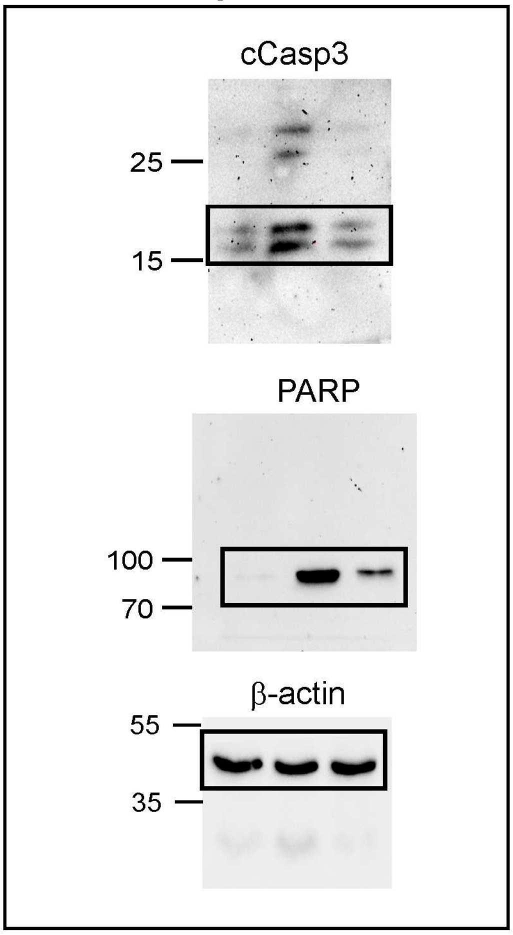

Figure 2H

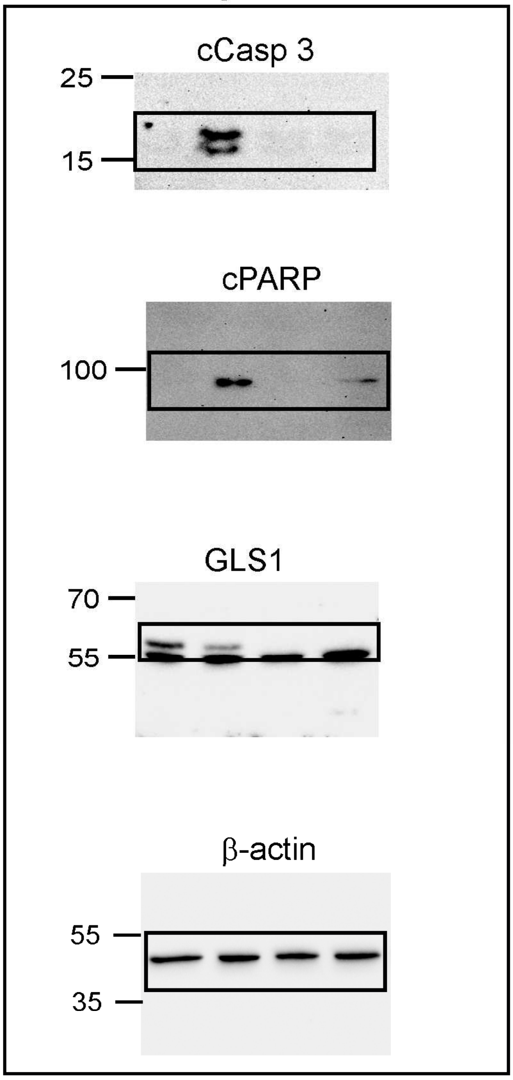

Figure 2I

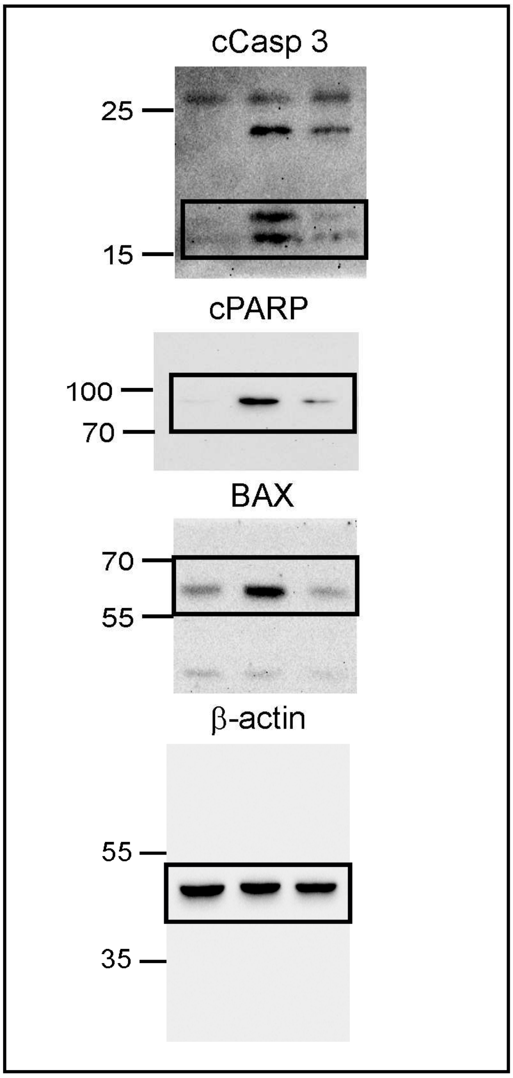

Sup. Fig. 2A

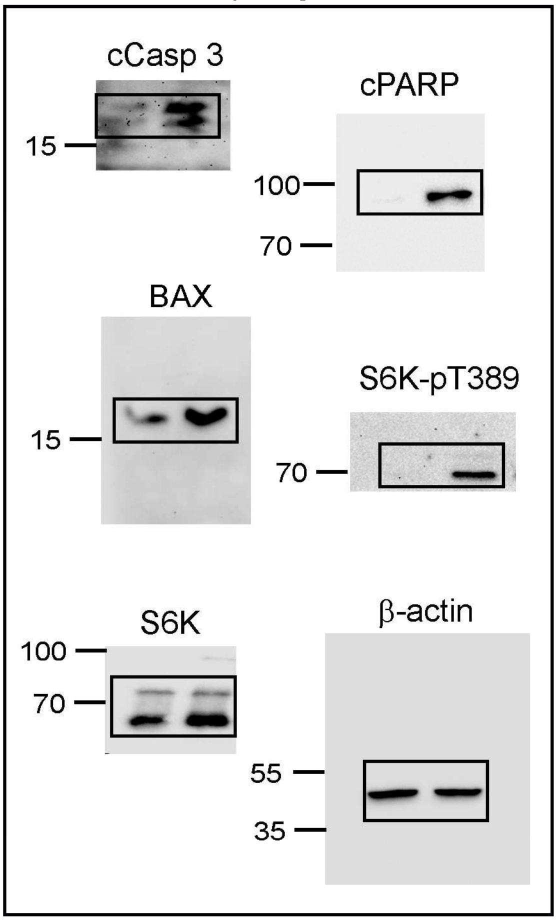

Sup. Fig. 2B

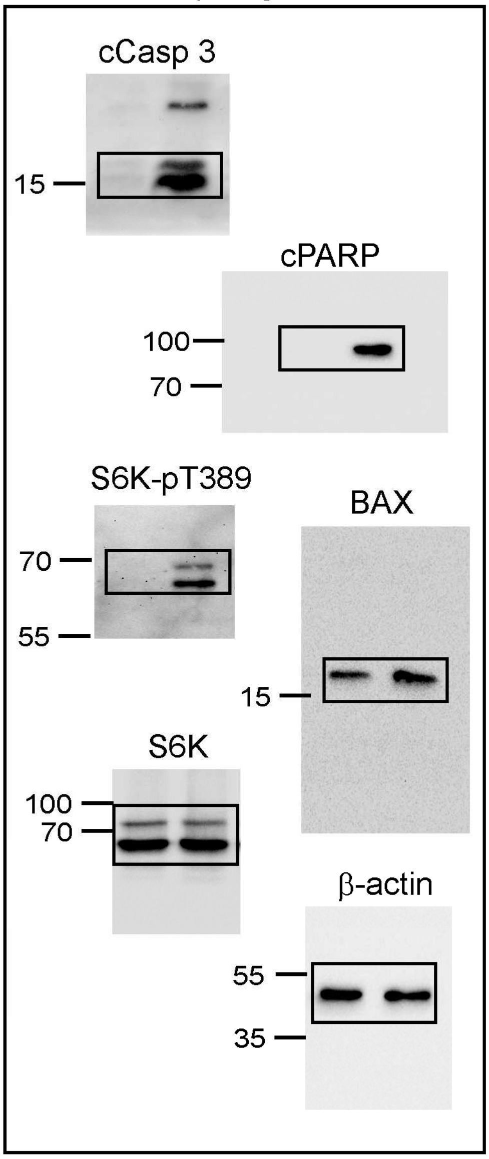

Sup. Fig. 2C

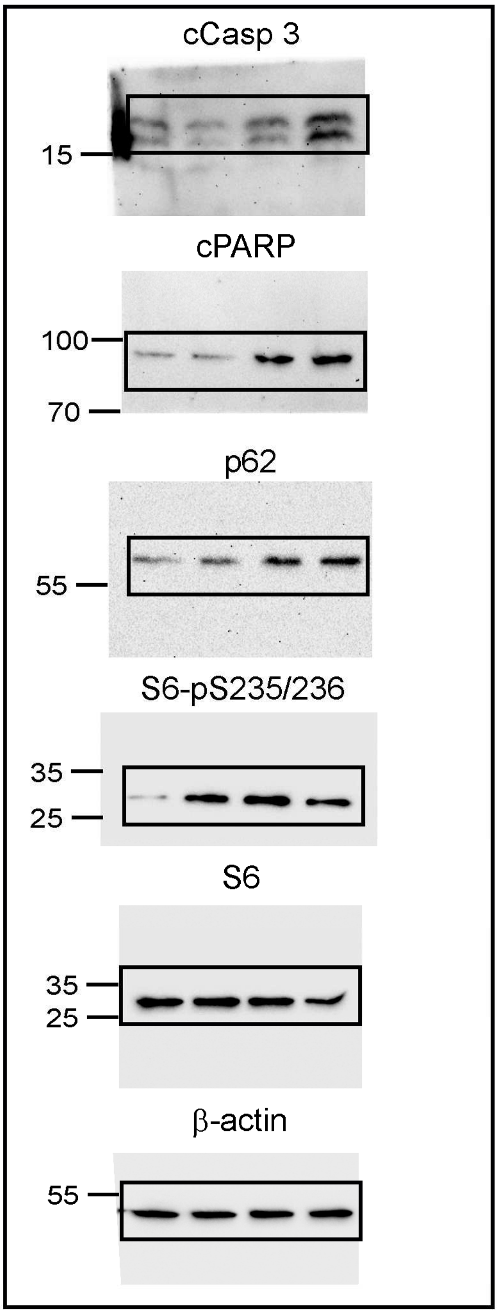

Sup. Fig. 2D

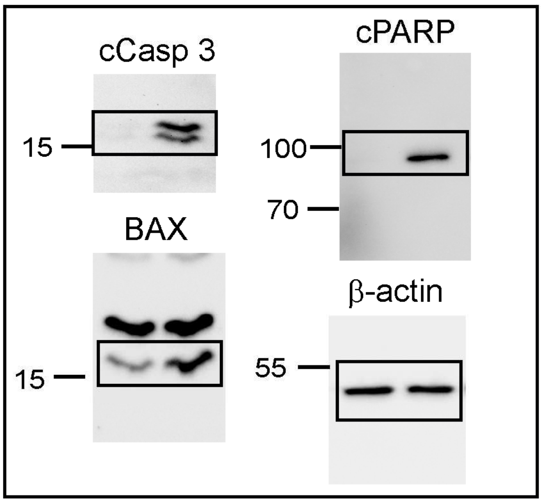

Sup. Fig. 2E

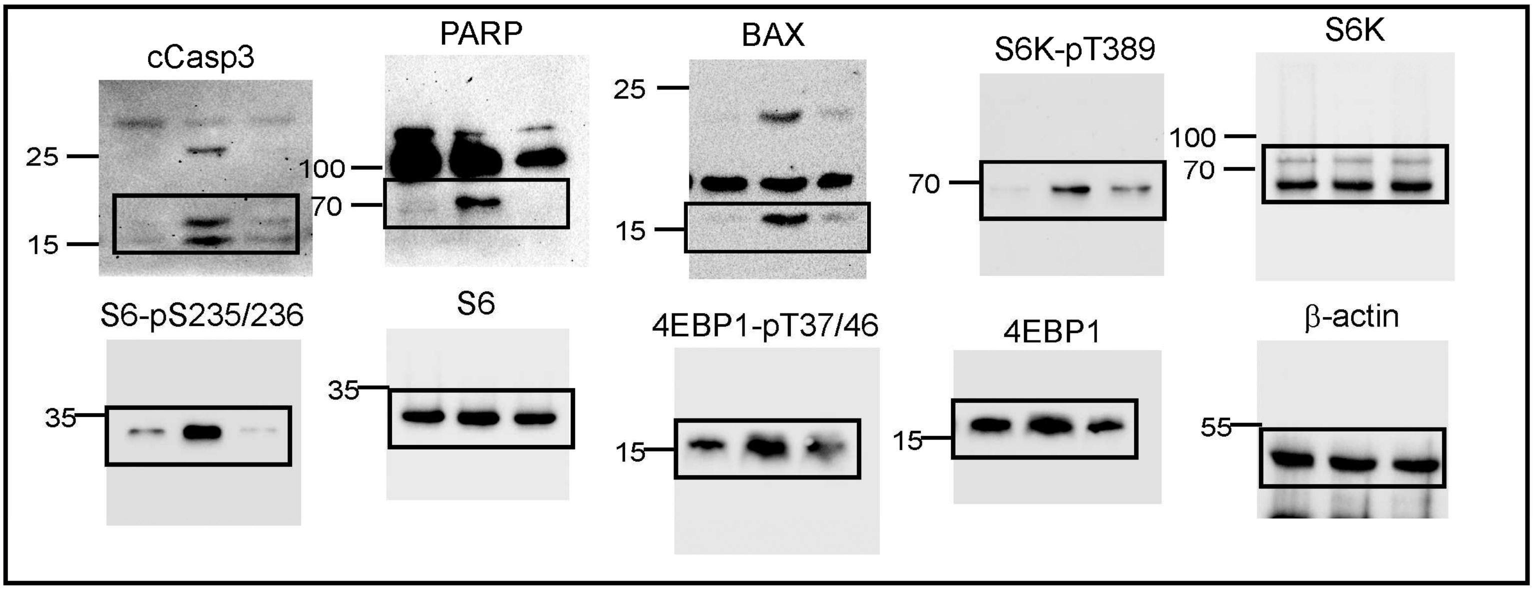

Sup. Fig. 2F

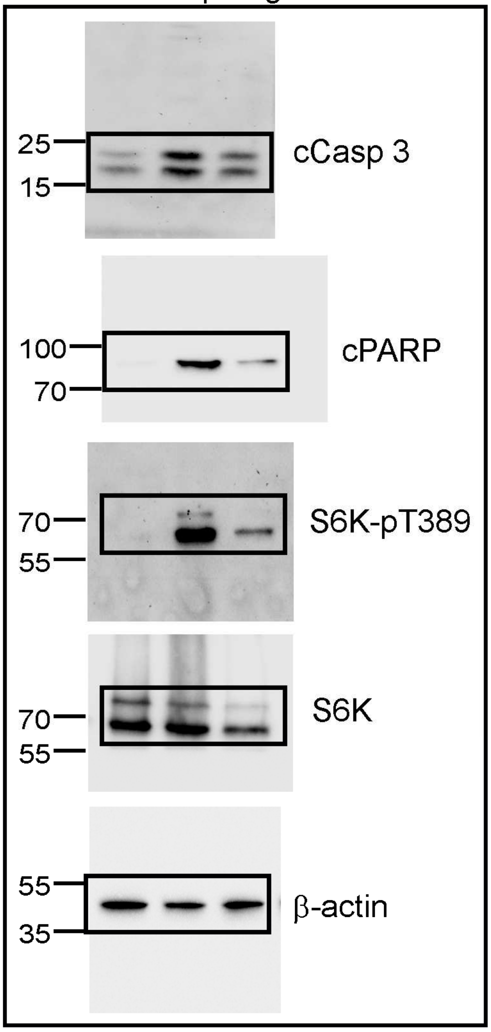

Sup. Fig. 2I

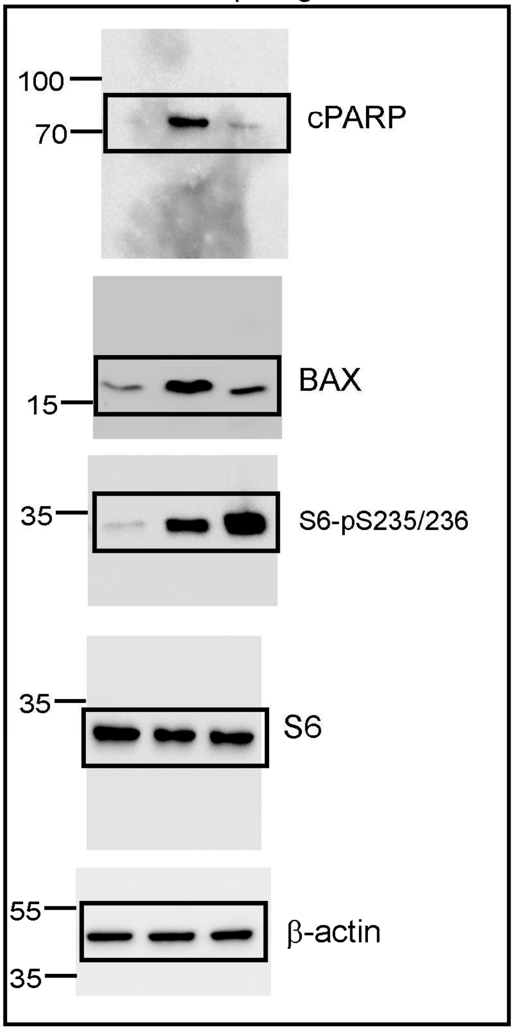

Sup. Fig. 2K

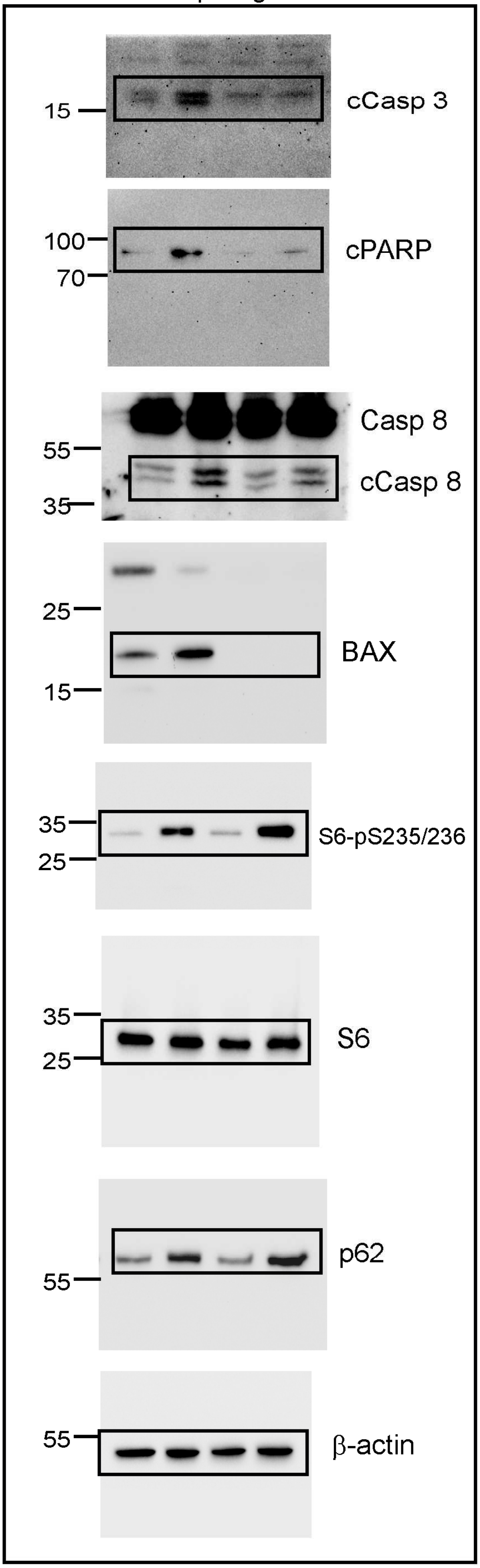

Sup. Fig. 2M

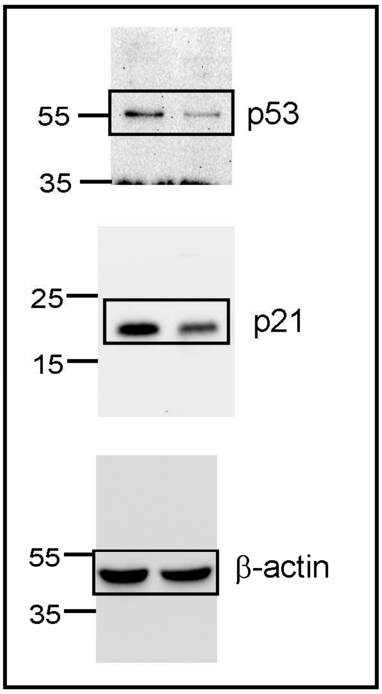

Sup. Fig. 2N

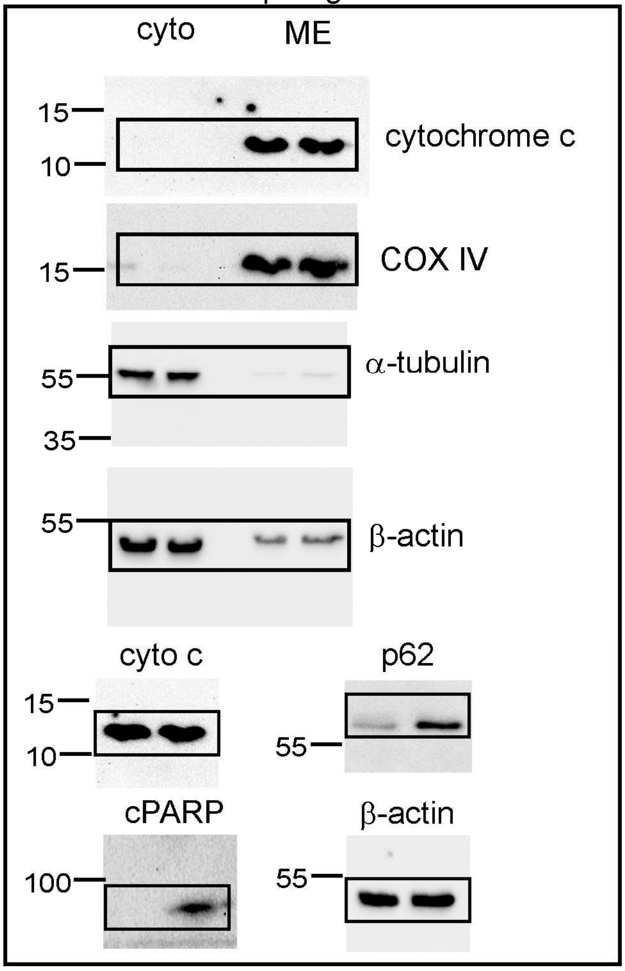

Figure 3D

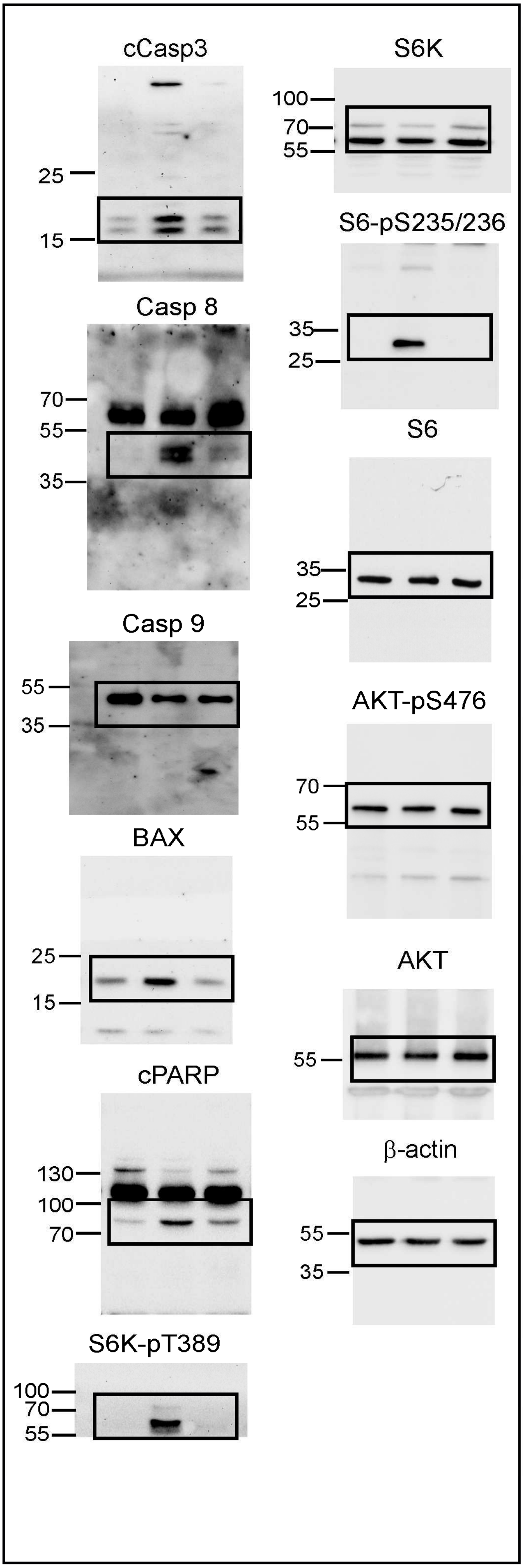

Figure 3E

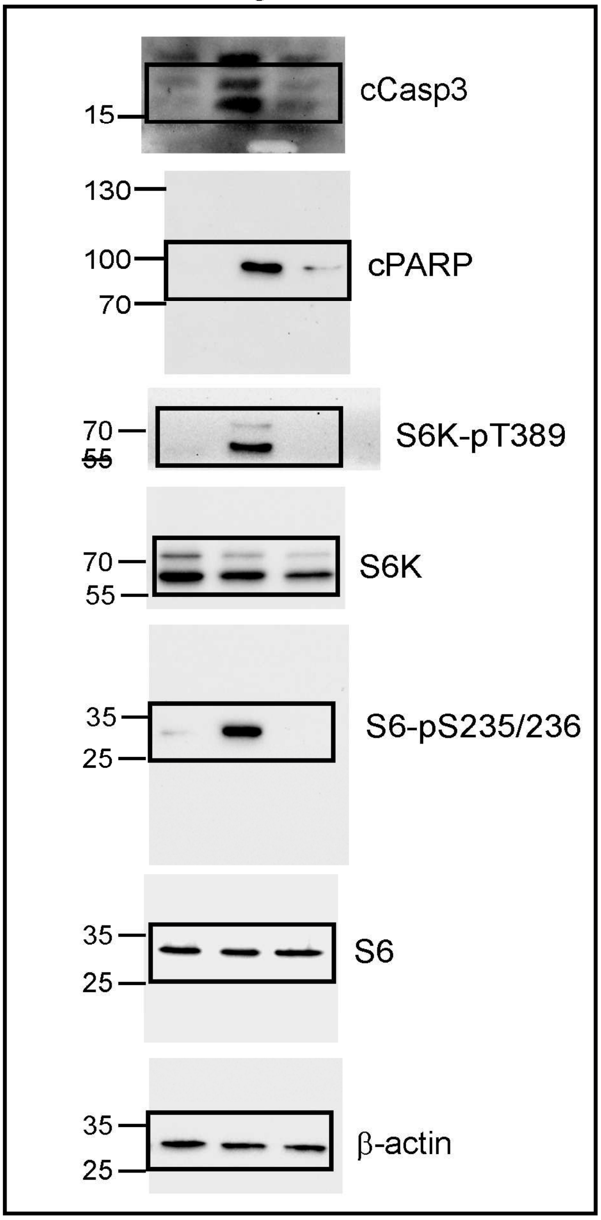

Figure 3F

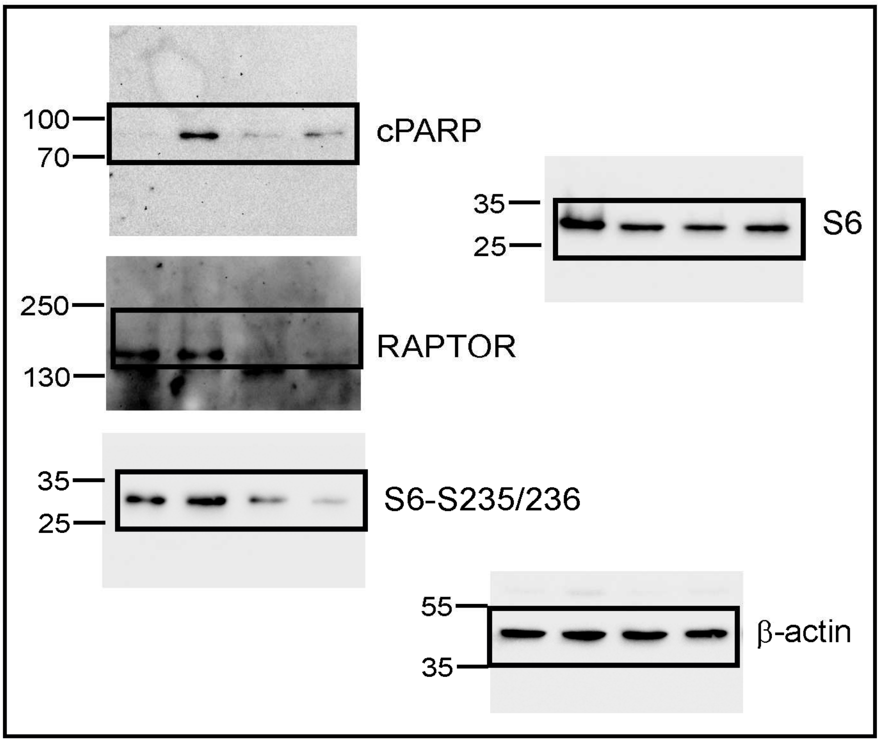

Sup. Fig. 3A

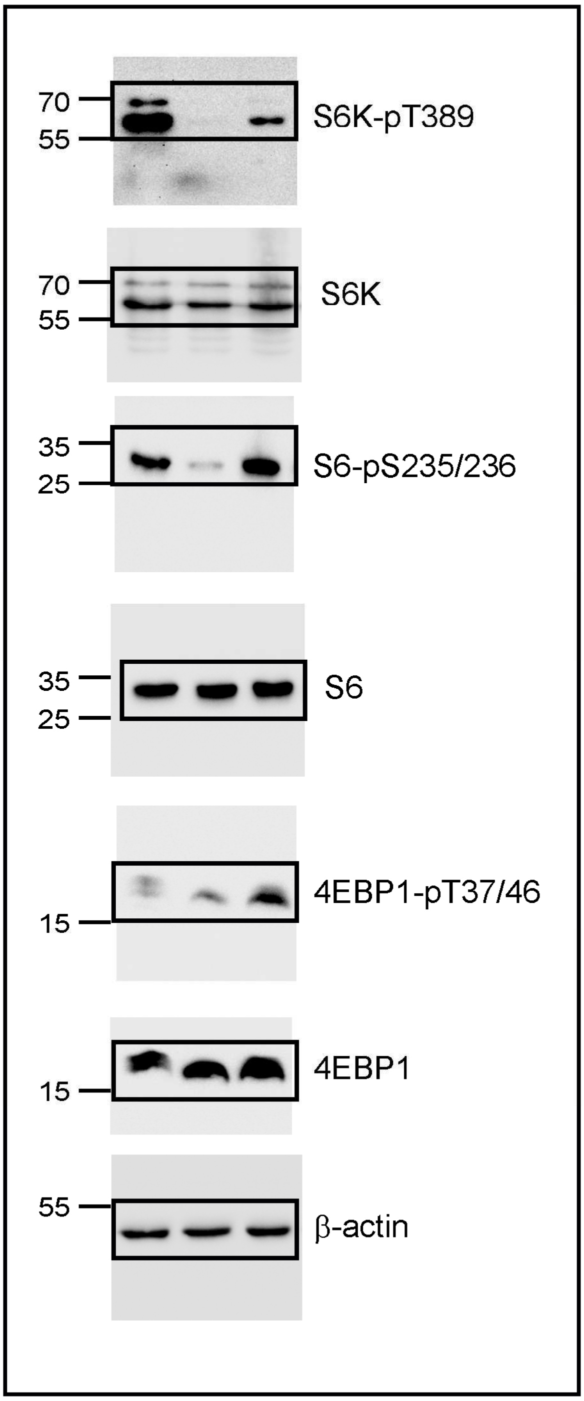

Sup. Fig. 3B

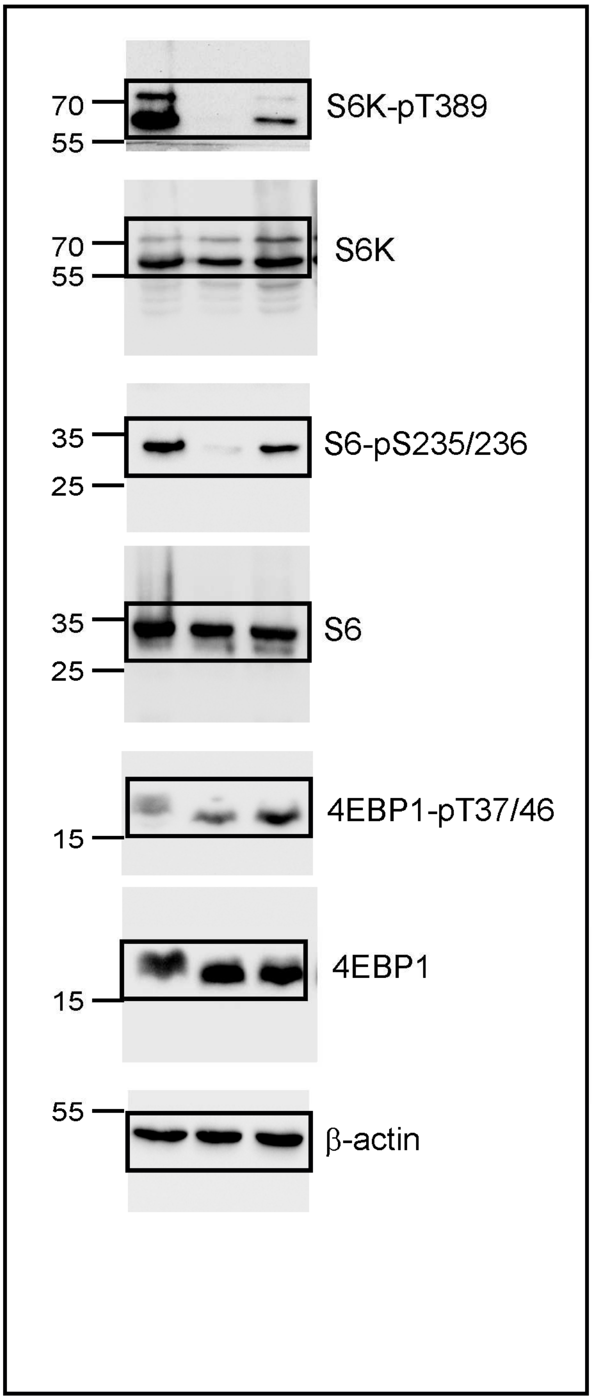

Sup. Fig. 3E

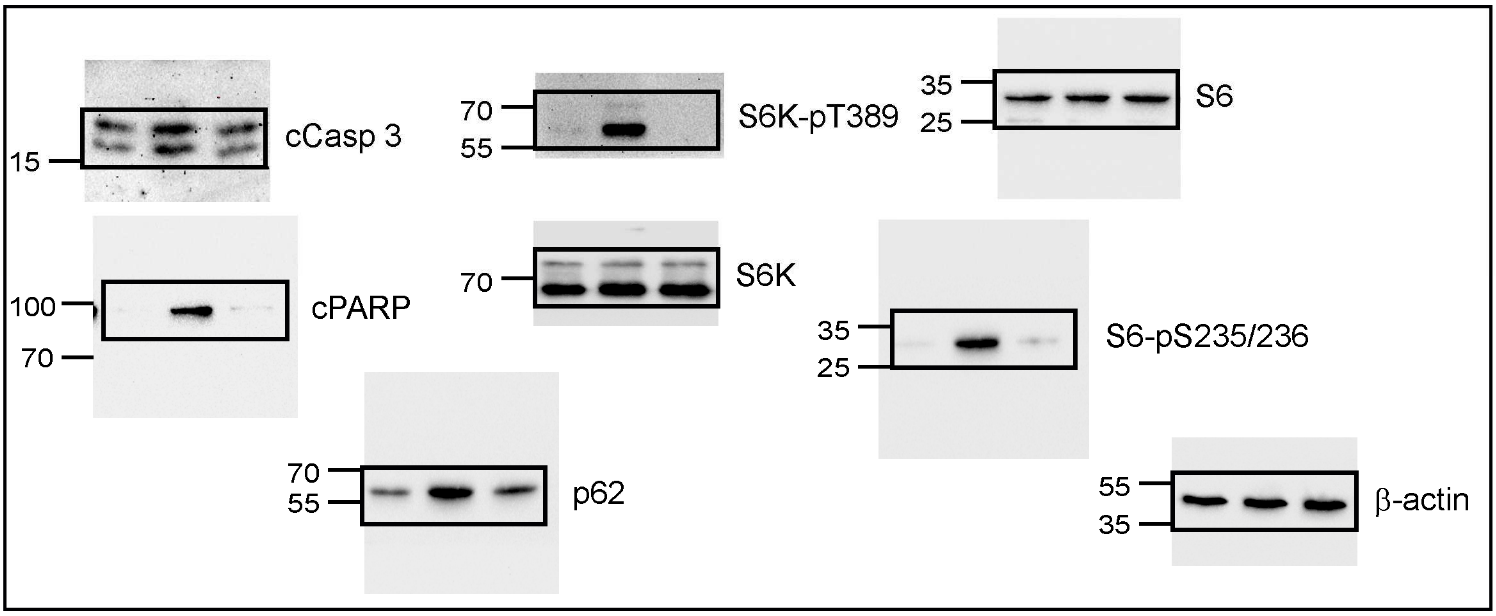

Sup. Fig. 3F

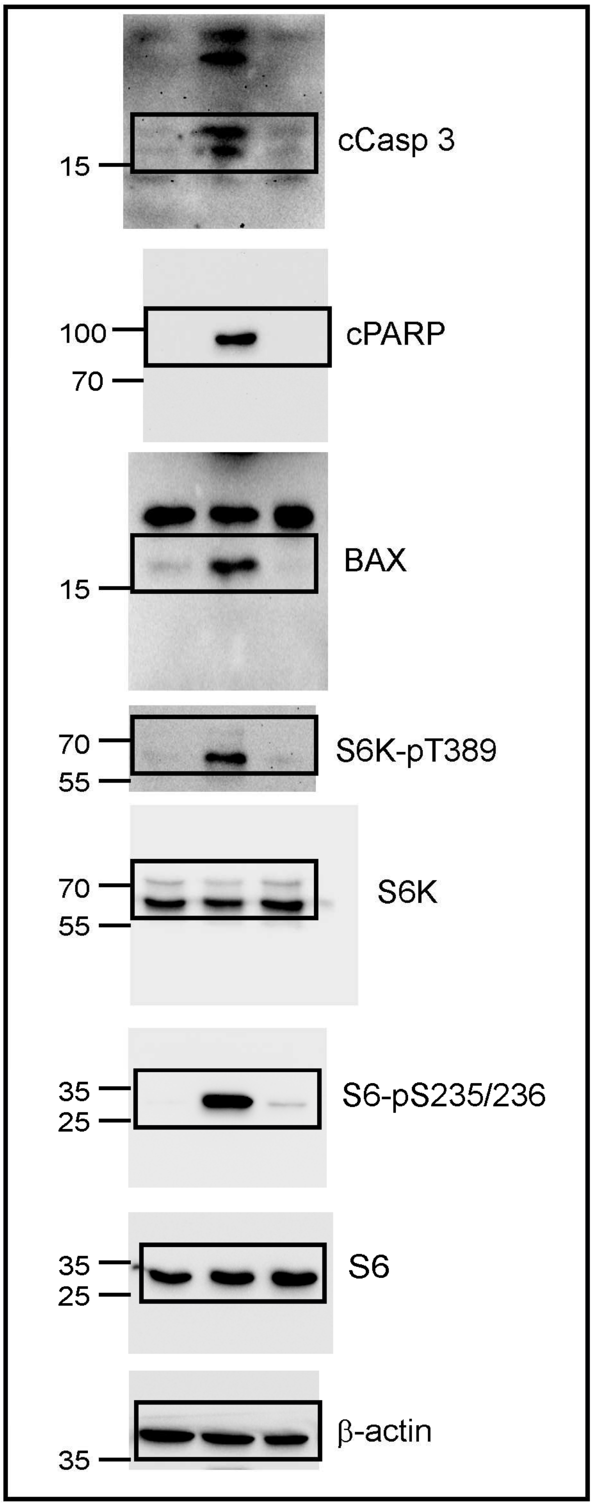

Sup. Fig. 3G

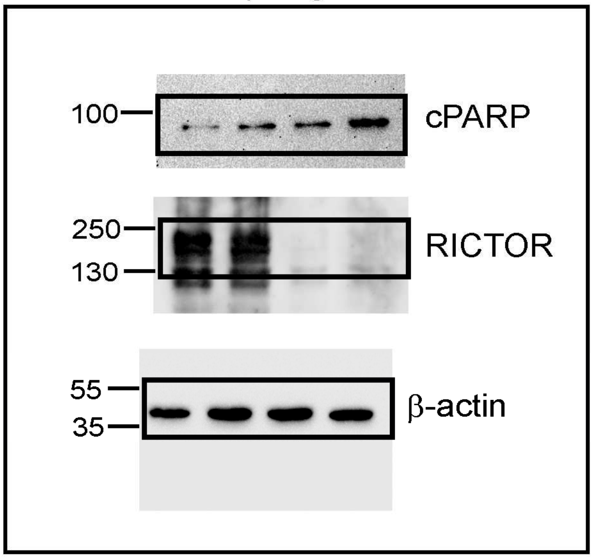

Sup. Fig. 3H

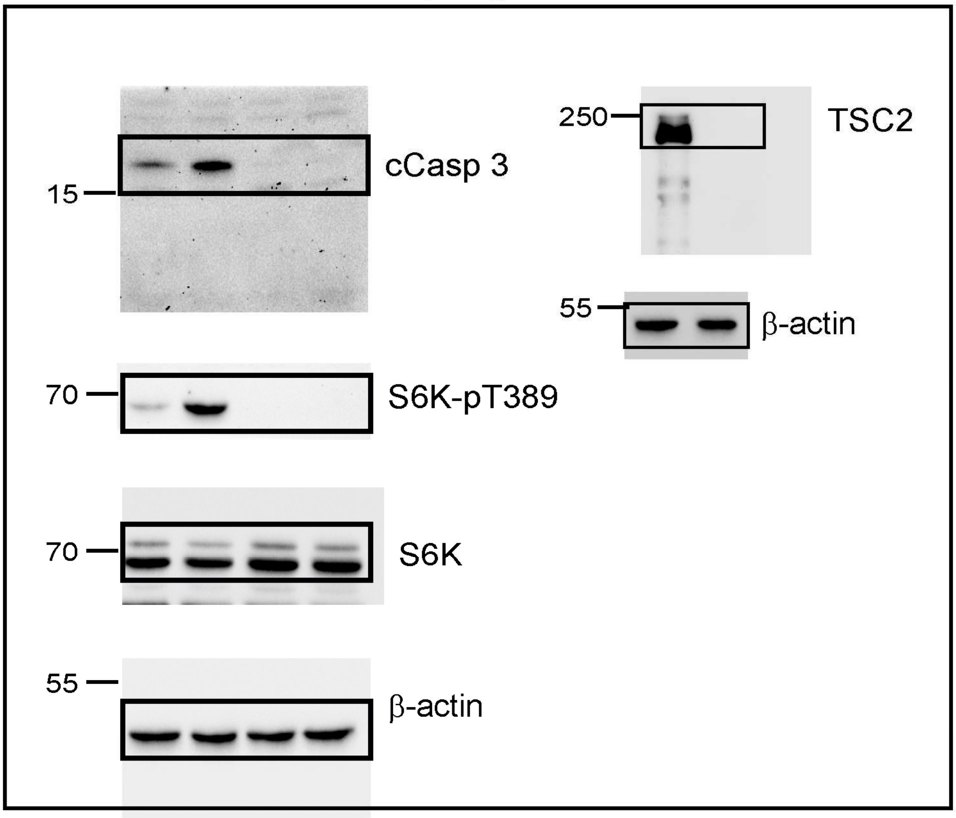

Sup. Fig. 3I

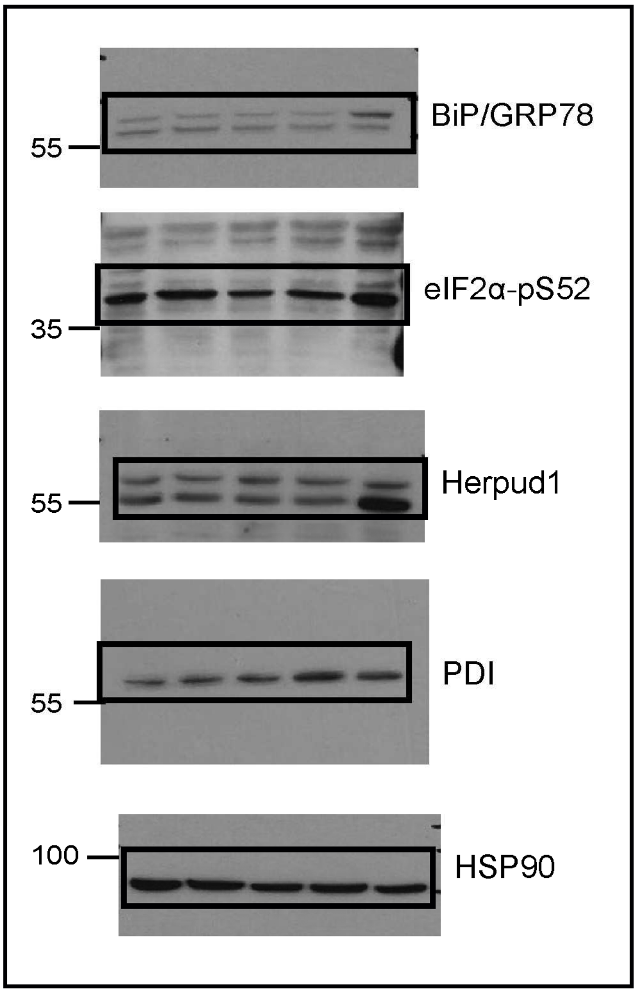

Sup. Fig. 3J

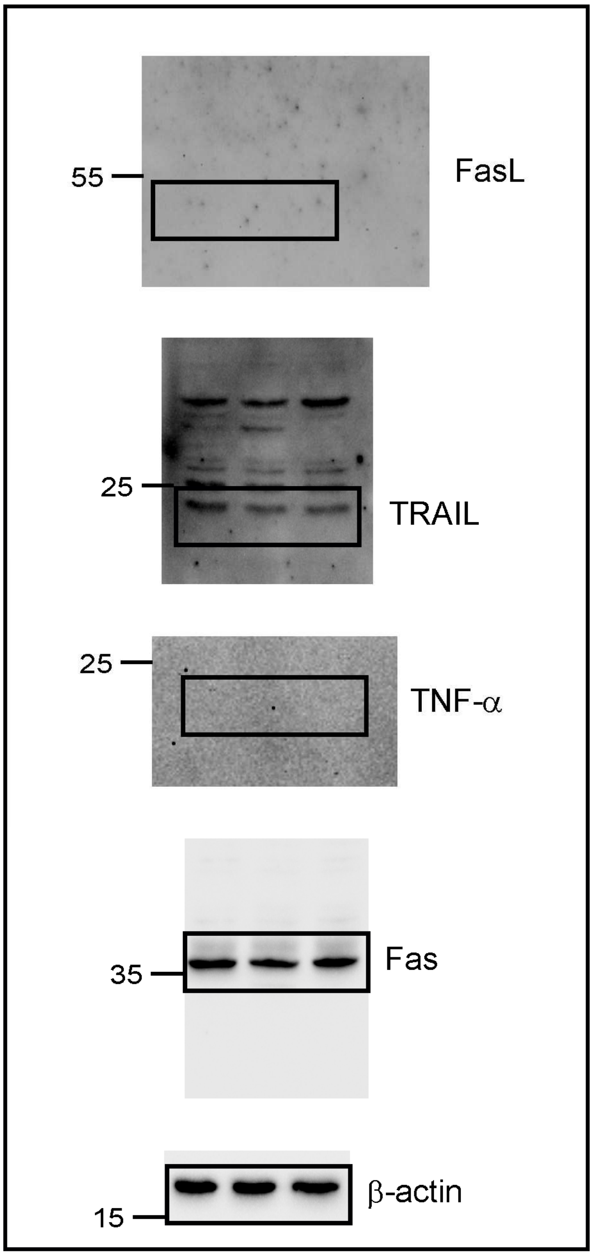

Sup. Fig. 3L

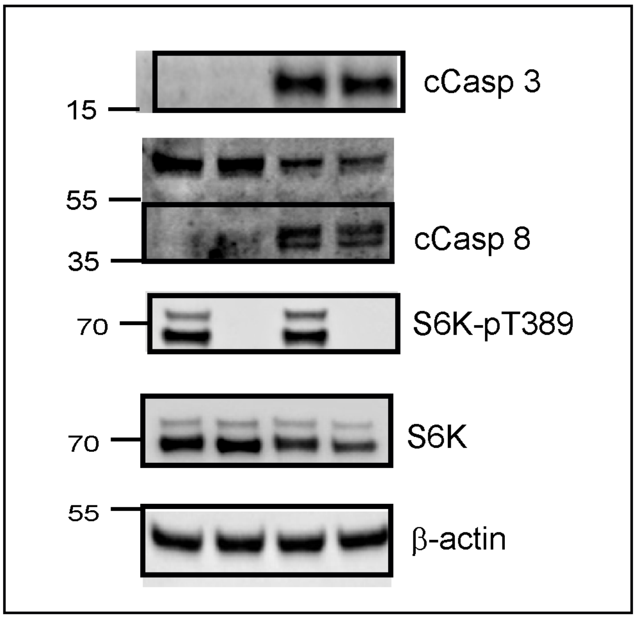

Sup. Fig. 3N

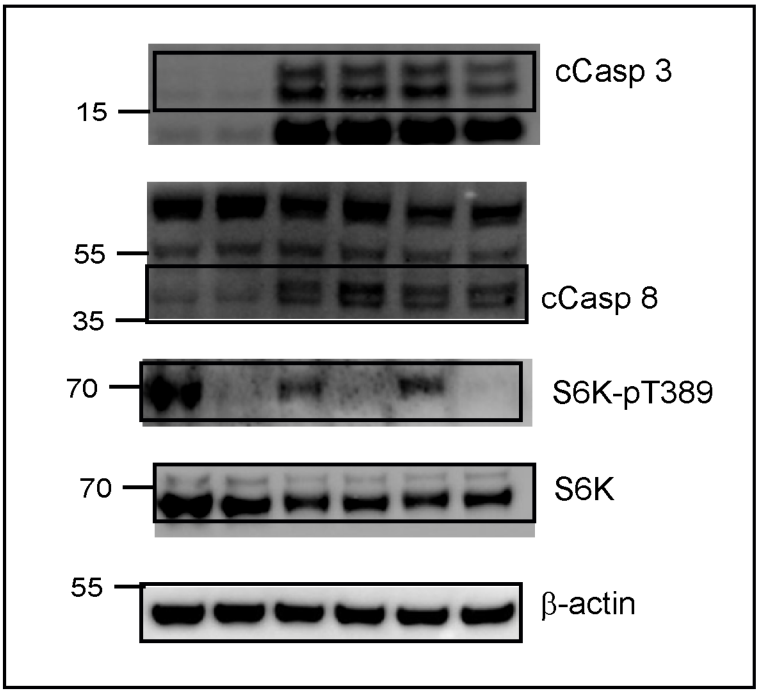

Figure 4B

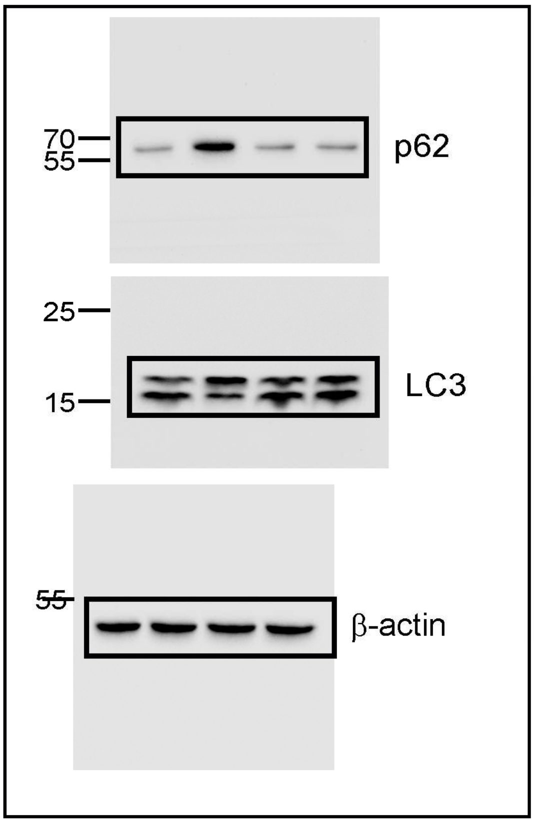

Figure 4C

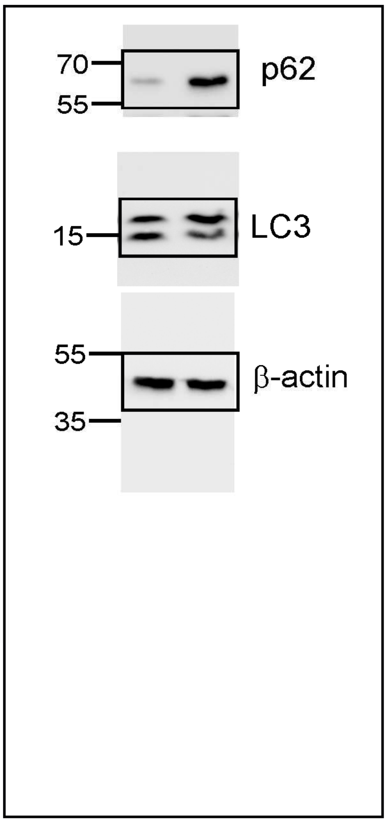

Figure 4G

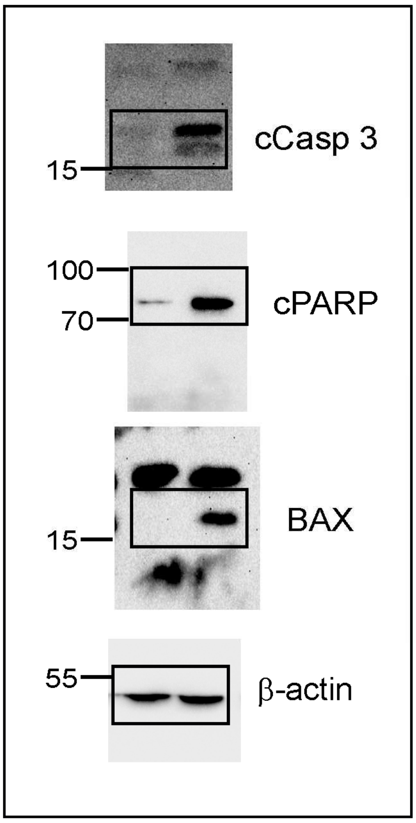

Figure 4I

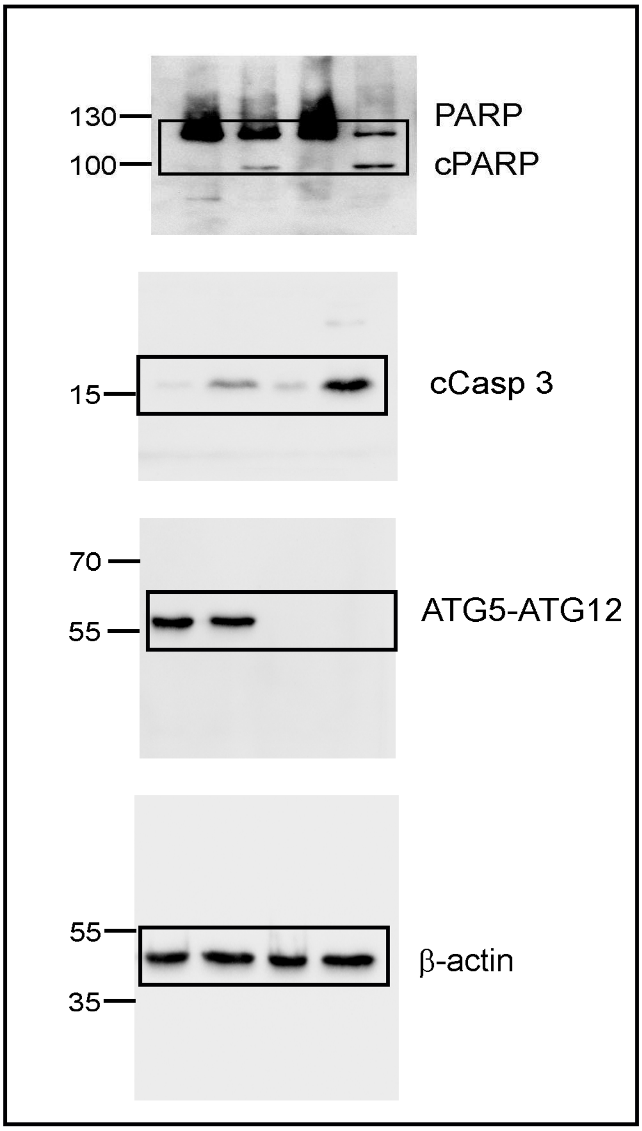

Sup. Fig. 4C

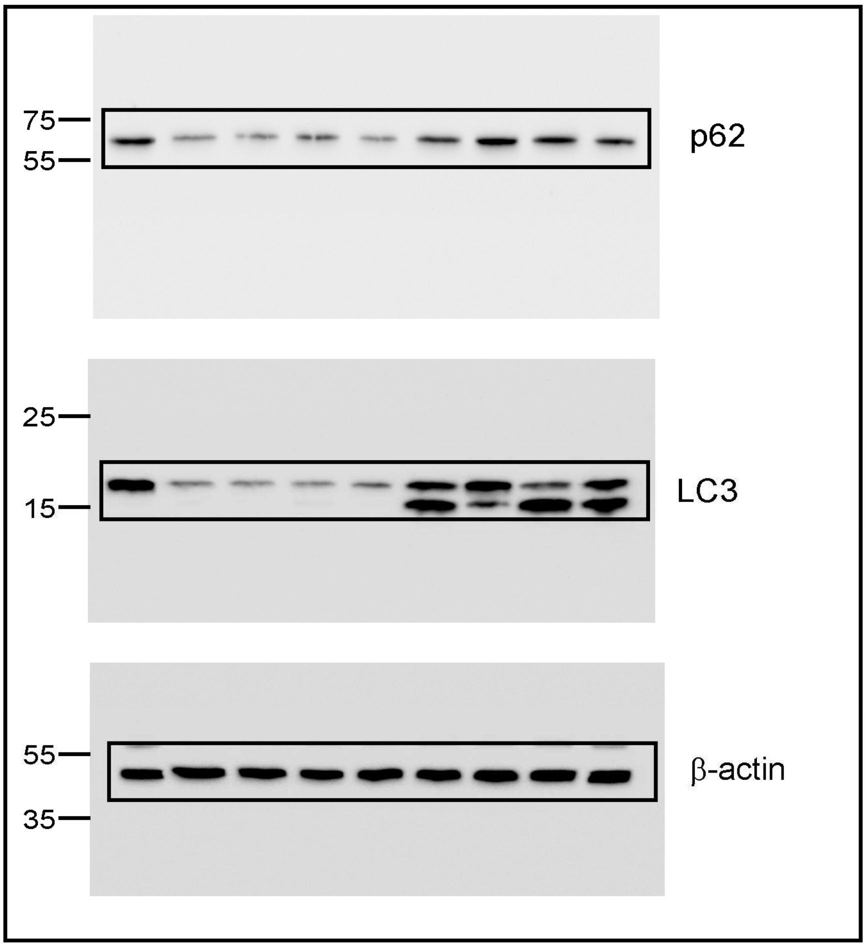

Sup. Fig. 4D

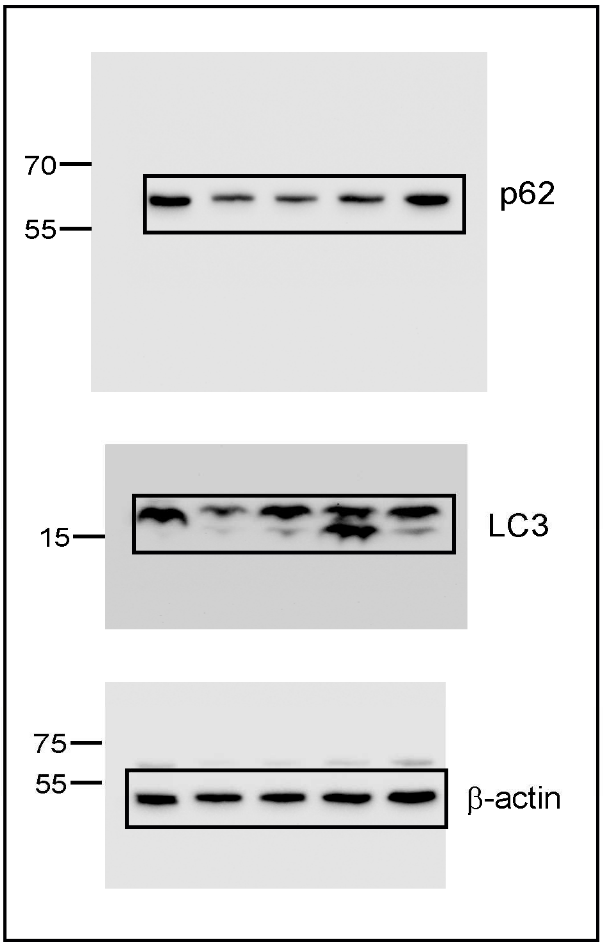

Sup. Fig. 4E

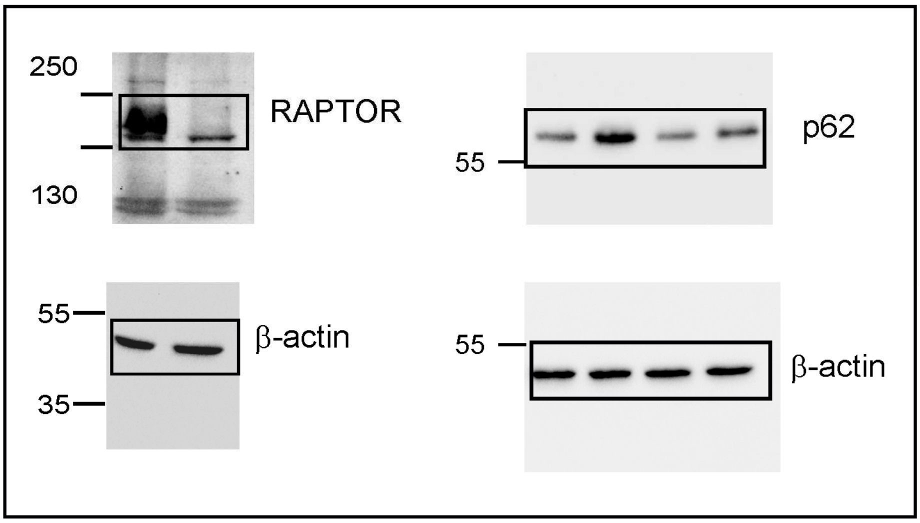

Sup. Fig. 4F

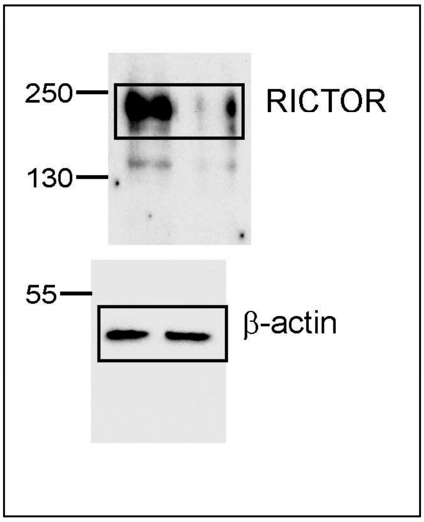

Sup. Fig. 4G

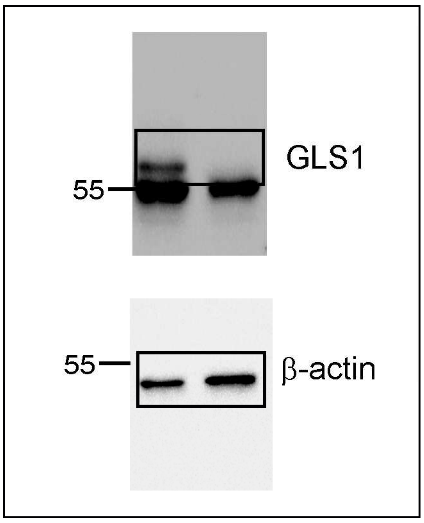

Sup. Fig. 4I

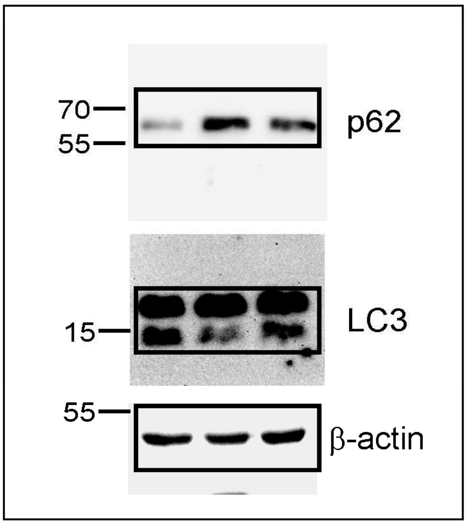

Sup. Fig. 4K

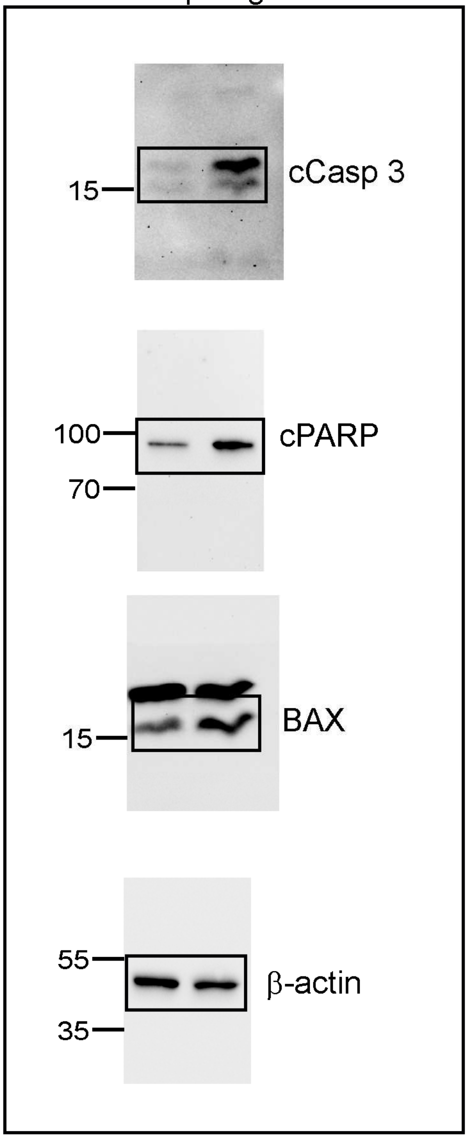

Figure 5D

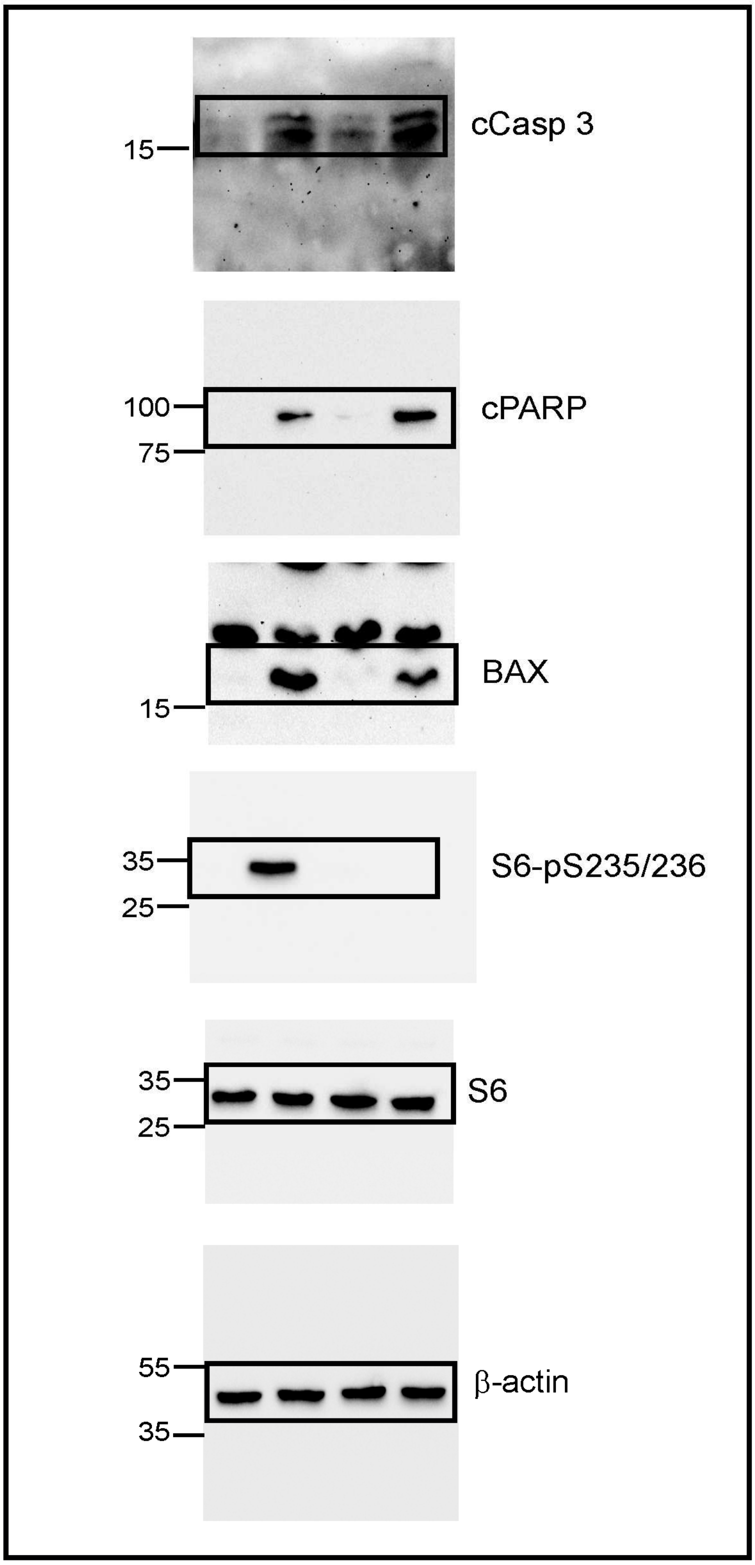

Figure 5H

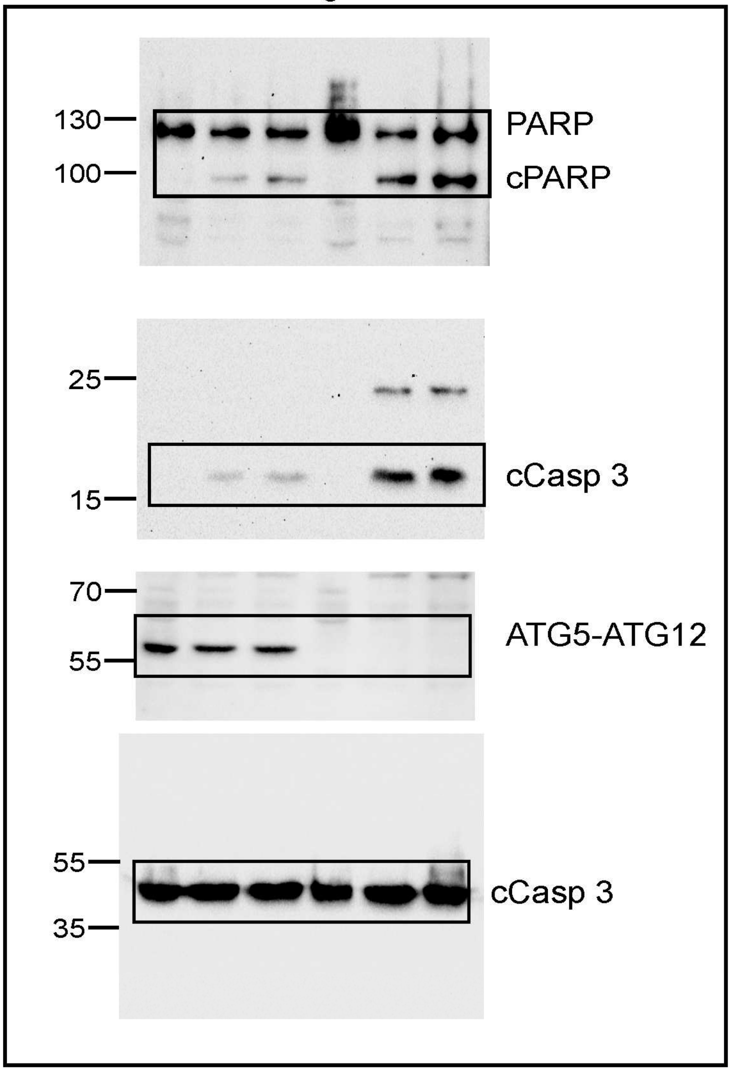

Sup. Fig. 5A

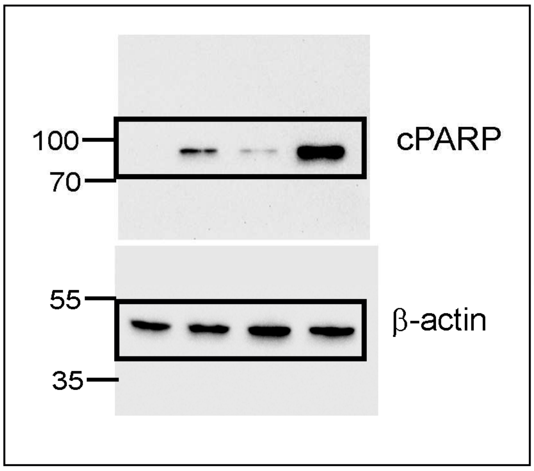

Sup. Fig. 5B

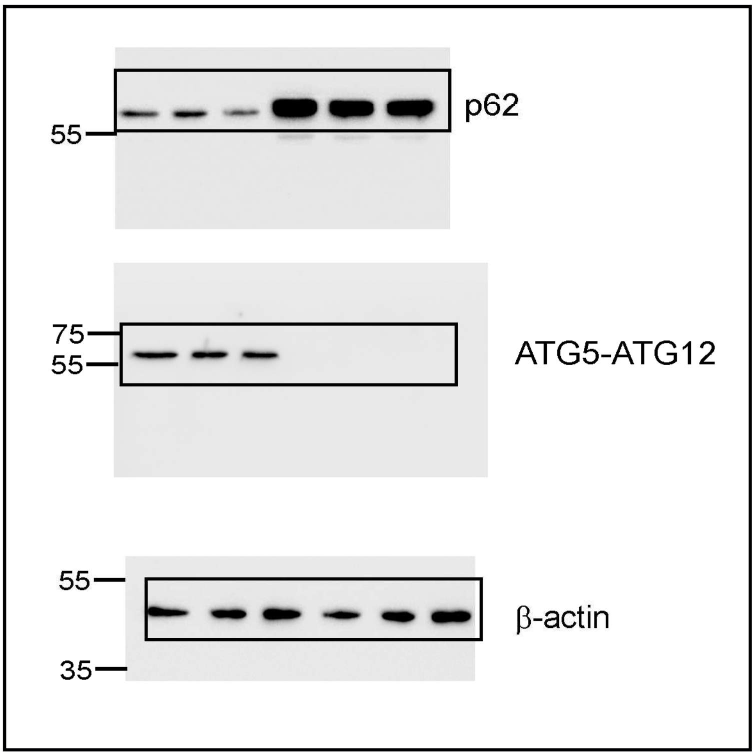

Sup. Fig. 5C

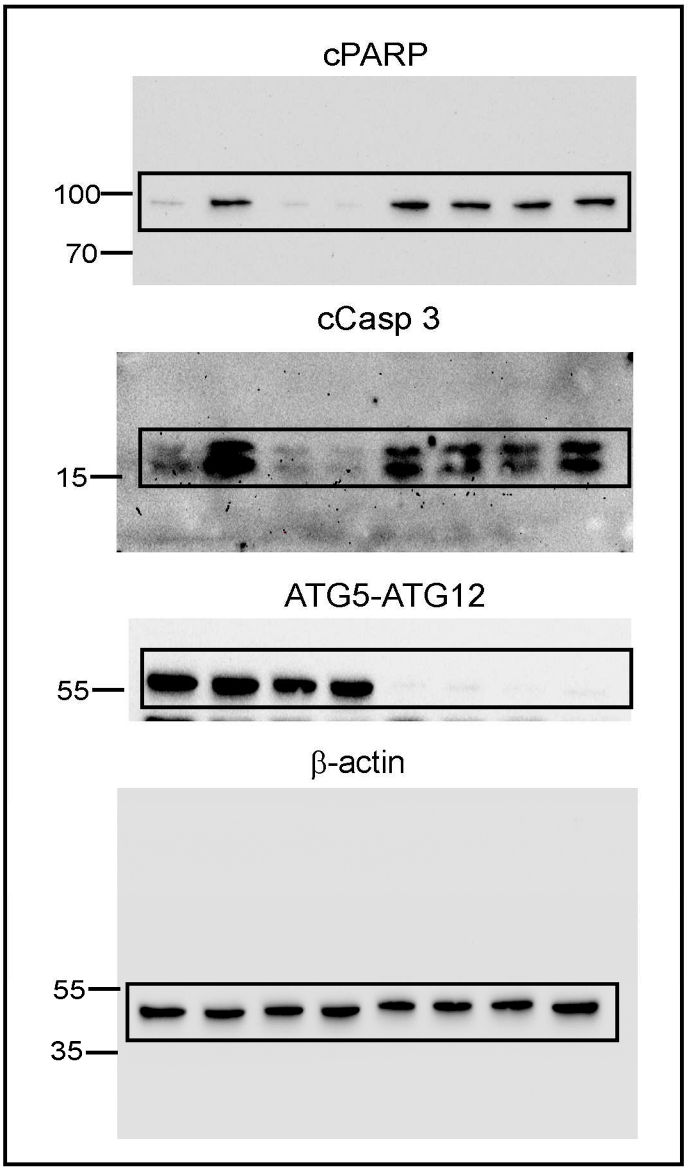

Sup. Fig. 5E

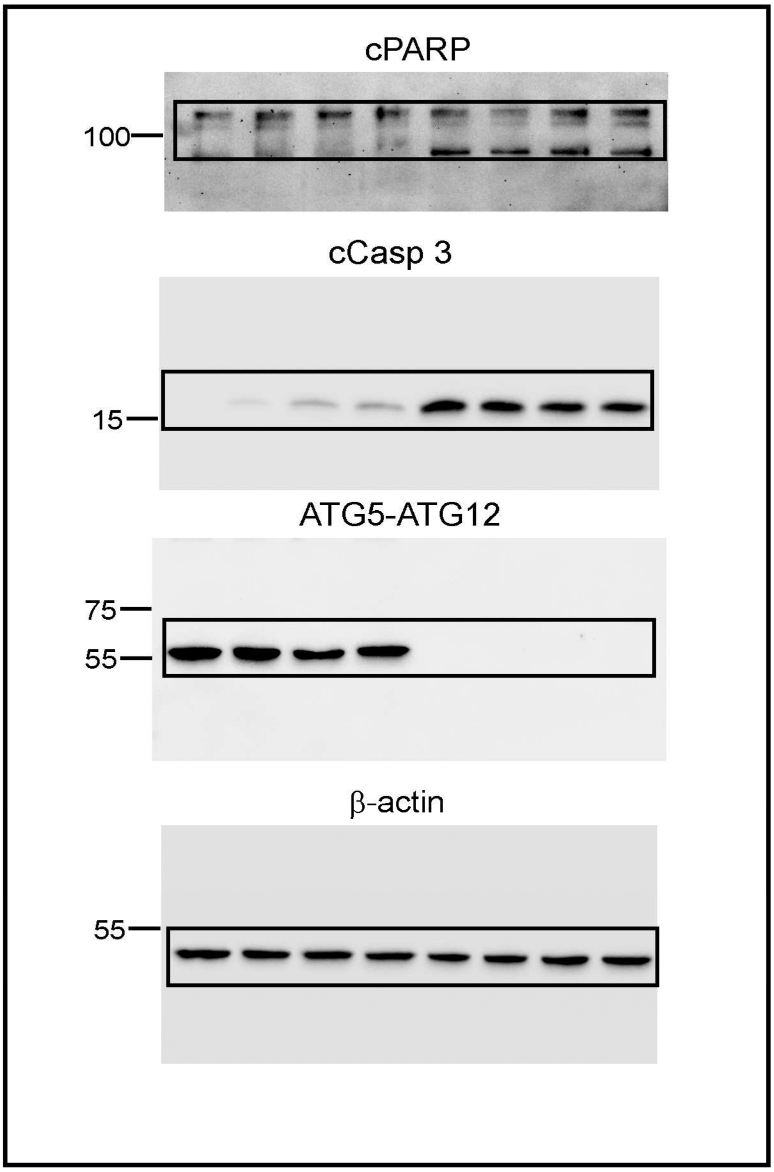

Fig. 6A

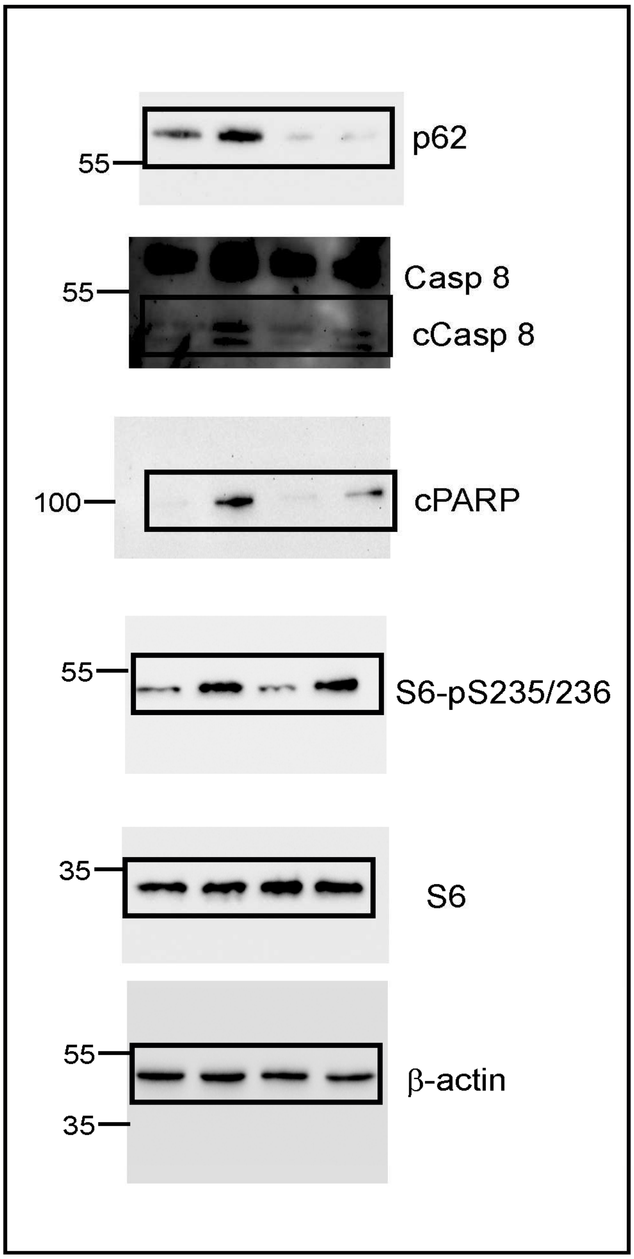

Fig. 6B

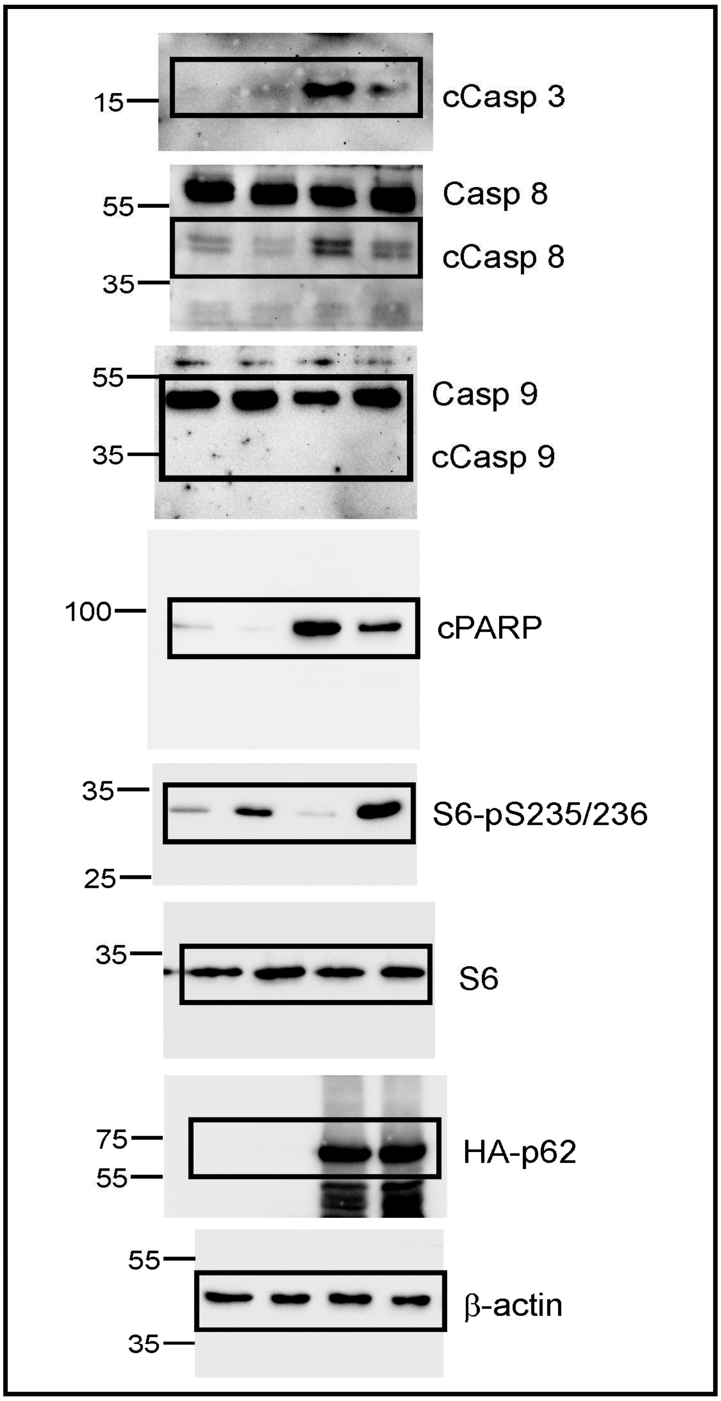

Fig. 6D

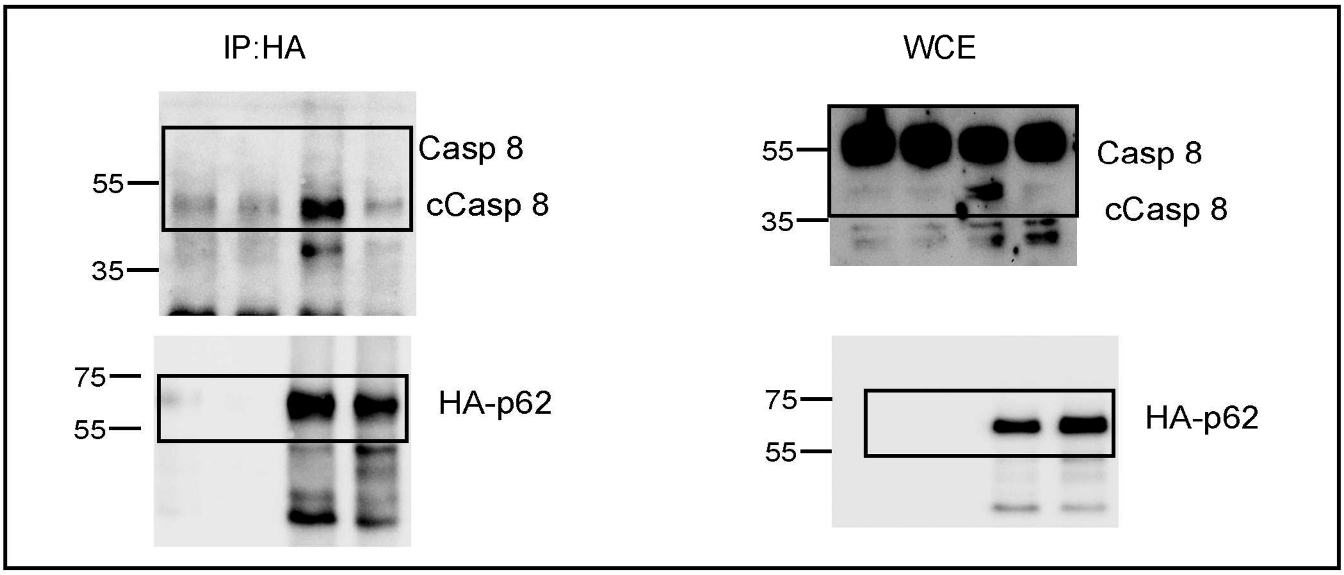

Supplementary Figure 6. Full blot images corresponding to wester blots shown in main and supplementary figures. In each image, the corresponding panel and antibody are indicated.
